# Molecular mechanisms underlying NLRP3 inflammasome activation and IL-1β production in air pollution fine particulate matter (PM_2.5_)-primed macrophages^☆^

**DOI:** 10.1016/j.envpol.2023.122997

**Published:** 2024-01-15

**Authors:** Lourdes Caceres, Tijani Abogunloko, Sara Malchow, Fabienne Ehret, Julian Merz, Xiaowei Li, Lucia Sol Mitre, Natalia Magnani, Deborah Tasat, Timothy Mwinyella, Lisa Spiga, Dymphie Suchanek, Larissa Fischer, Oliver Gorka, Mark Colin Gissler, Ingo Hilgendorf, Peter Stachon, Eva Rog-Zielinska, Olaf Groß, Dirk Westermann, Pablo Evelson, Dennis Wolf, Timoteo Marchini

**Affiliations:** a Department of Cardiology and Angiology, University Heart Center, University of Freiburg, 79106, Freiburg im Breisgau, Germany; b Faculty of Medicine, University of Freiburg, 79110, Freiburg im Breisgau, Germany; c Universidad de Buenos Aires, Facultad de Farmacia y Bioquímica, Departamento de Ciencias Químicas, C1113AAD, Buenos Aires, Argentina; d CONICET – Universidad de Buenos Aires, Instituto de Bioquímica y Medicina Molecular Prof. Alberto Boveris (IBIMOL), C1113AAD, Buenos Aires, Argentina; e Spemann Graduate School of Biology and Medicine (SGBM), University of Freiburg, 79104, Freiburg, Germany; f Faculty of Biology, University of Freiburg, 79104, Freiburg im Breisgau, Germany; g Universidad Nacional de General San Martín, Escuela de Ciencia y Tecnología, B1650, General San Martín, Argentina; h Institute of Neuropathology, Faculty of Medicine, University of Freiburg, 79106, Freiburg, Germany; i Institute for Experimental Cardiovascular Medicine, University Heart Center, Faculty of Medicine, University of Freiburg, 79110, Freiburg im Breisgau, Germany

**Keywords:** Particulate matter, Macrophages, Inflammation, Lysosomal disruption, K^+^ efflux, Mitochondria

## Abstract

Exposure to air pollution fine particulate matter (PM_2.5_) aggravates respiratory and cardiovascular diseases. It has been proposed that PM_2.5_ uptake by alveolar macrophages promotes local inflammation that ignites a systemic response, but precise underlying mechanisms remain unclear. Here, we demonstrate that PM_2.5_ phagocytosis leads to NLRP3 inflammasome activation and subsequent release of the pro-inflammatory master cytokine IL-1β. Inflammasome priming and assembly was time- and dose-dependent in inflammasome-reporter THP-1-ASC-GFP cells, and consistent across PM_2.5_ samples of variable chemical composition. While inflammasome activation was promoted by different PM_2.5_ surrogates, significant IL-1β release could only be observed after stimulation with transition-metal rich Residual Oil Fly Ash (ROFA) particles. This effect was confirmed in primary human monocyte-derived macrophages and murine bone marrow-derived macrophages (BMDMs), and by confocal imaging of inflammasome-reporter ASC-Citrine BMDMs. IL-1β release by ROFA was dependent on the NLRP3 inflammasome, as indicated by lack of IL-1β production in ROFA-exposed NLRP3-deficient (*Nlrp3*^*−/−*^) BMDMs, and by specific NLRP3 inhibition with the pharmacological compound MCC950. In addition, while ROFA promoted the upregulation of pro-inflammatory gene expression and cytokines release, MCC950 reduced TNF-α, IL-6, and CCL2 production. Furthermore, inhibition of TNF-α with a neutralizing antibody decreased IL-1β release in ROFA-exposed BMDMs. Using electron tomography, ROFA particles were observed inside intracellular vesicles and mitochondria, which showed signs of ultrastructural damage. Mechanistically, we identified lysosomal rupture, K^+^ efflux, and impaired mitochondrial function as important prerequisites for ROFA-mediated IL-1β release. Interestingly, specific inhibition of superoxide anion production (O_2_^•-^) from mitochondrial respiratory Complex I, but not III, blunted IL-1β release in ROFA-exposed BMDMs. Our findings unravel the mechanism by which PM_2.5_ promotes IL-1β release in macrophages and provide a novel link between innate immune response and exposure to air pollution PM_2.5_.

## Highlights

• Incubation with PM_2.5_ promotes IL-1β release in murine and human macrophages.
• PM_2.5_-induced IL-1β release is blocked in *Nlrp3*^−/−^ macrophages and by MCC950.
• PM_2.5_ uptake triggers both NLRP3 inflammasome priming and assembly.
• Lysosomal rupture and K^+^ efflux mediate IL-1β release following PM_2.5_ uptake.
• O_2_^•-^ production from Complex I, but not III, drives PM_2.5_-induced IL-1β release.

## Graphical abstract

**Figure:**
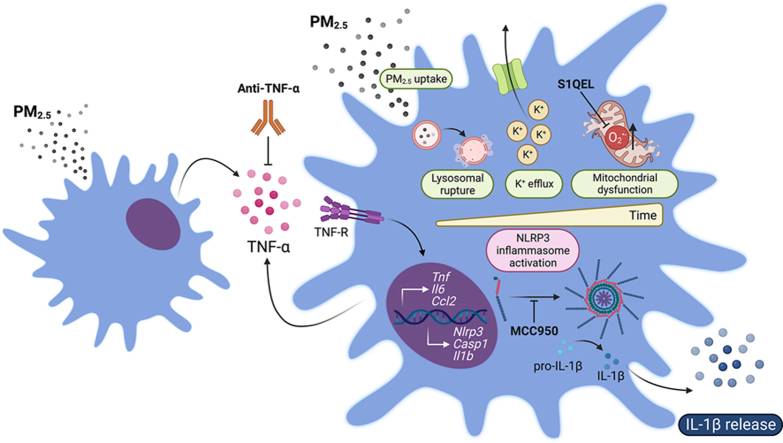

## List of abbreviations

ASC: Apoptosis-associated speck-like protein containing a CARD
BMDMs: Bone marrow-derived macrophages
CAPs: Concentrated Air Particles
NLRP3: NOD- LRR- and pyrin domain-containing protein 3
O_2_^•-^: Superoxide anion
PBMCs: Peripheral blood mononuclear cells
PM_2.5_: Fine particulate matter
ROFA: Residual Oil Fly Ash
SRM: Standard Reference Material

## 1 Introduction

Air pollution is one of the leading drivers of global mortality (Collaborators, 2020). Despite ongoing efforts to improve air quality, deaths associated with air pollution continue to rise as a consequence of increasing industrialization and urbanization (Fuller et al., 2022). It is estimated that air pollution-related mortality is mainly attributable to cardiovascular diseases, such as myocardial infarction and stroke, and respiratory conditions including chronic obstructive pulmonary disease (COPD), respiratory infections, and lung cancer (Brook et al., 2010; Lelieveld et al., 2019; Pope et al., 2020).

Although air pollution is a complex mixture of toxic gaseous, liquid, and solid components, numerous epidemiological studies indicate that fine particulate matter (PM_2.5_) is the most hazardous air pollutant (Al-Kindi et al., 2020; Brook et al., 2010). PM_2.5_ arise from fossil fuel combustion during power generation and industrial processes, and from diesel exhaust in urban areas (Bai et al., 2018). An innate pulmonary and systemic immune response mediated by pro-inflammatory cytokine release from alveolar macrophages is a hallmark of PM_2.5_ pathogenic mechanisms (Marchini et al., 2020). However, the molecular pathways linking PM_2.5_ exposure and pro-inflammatory cytokine release remain unclear.

Inflammasomes are multi-protein intracellular complexes that mediate inflammation in response to a wide range of danger signals including Pathogen-Associated Molecular Patterns (PAMPs) and Danger-Associated Molecular Patterns (DAMPs) (Kelley et al., 2019). The main role of inflammasomes is Caspase-1-mediated cleavage of pro-IL-1β into its biologically active form IL-1β. Likewise, Caspase-1 also processes the inactive precursor of IL-18 to generate mature IL-18. NLRP3 inflammasome activation is a two-step process. First, the priming step takes place when the inflammasome proteins NLRP3, Caspase-1, pro-IL-1β and pro-IL-18 are upregulated after recognition of PAMPs and DAMPs by Toll-like receptors (TLRs), TNF-α, and Nuclear Factor-κB (NF-κB) signalling. Second, the NLRP3 inflammasome is activated by a multiplicity of stimuli, which down-stream result in the recruitment of the apoptosis-associated speck-like protein containing a CARD (ASC) adaptor protein, formation of ASC-specks, and Caspase-1 mediated cleavage of IL-1β and IL-18. This event encompasses multiple signals that are not mutually exclusive, including efflux of potassium (K^+^), lysosomal disruption, and mitochondrial dysfunction (Swanson et al., 2019). As IL-1β is a key driver of pulmonary (De Nardo et al., 2014) and cardiovascular diseases (Silvis et al., 2021), the inflammasomes have therefore emerged as novel pharmacological targets (Olsen et al., 2022; Ridker et al., 2017). This might be best exemplified by the discovery of MCC950, a small molecule inhibitor that specifically blocks NLRP3 oligomerization and IL-1β release (Coll et al., 2019).

After inhalation, alveolar macrophages engulf PM_2.5_ and release pro-inflammatory cytokines including TNF-α, IL-6, and CCL2, which eventually boost the infiltration of circulating myeloid cells into the alveolar space and enhance subsequent tissue damage (Marchini et al., 2022). Previous reports have suggested that the exposure to various particulate stimuli, such as silica, asbestos, monosodium urate, and cholesterol crystals, triggers NLRP3 inflammasome activation and IL-1β release (Shirasuna et al., 2019). Recent findings also suggest that NLRP3-dependent release of IL-1β upon exposure to PM_2.5_ contributes to the development of lung fibrosis (Cao et al., 2022; Zheng et al., 2018) and cancer (Hill et al., 2023), cardiovascular injury (Du et al., 2019; Duan et al., 2019), and insulin resistance (Zhong et al., 2023). However, the specific molecular pathways that promote NLRP3 activation in response to PM_2.5_ have not been comprehensively addressed. In this study, we aim to unravel the mechanisms involved in NLRP3 inflammasome activation and IL-1β release by PM_2.5_ in human and mouse monocytes and macrophages.

## 2 Materials and Methods

An expanded Materials and Methods section is available in the Data Supplement.

### 2.1 PM_2.5_ samples

Residual Oil Fly Ash (ROFA), Concentrated Ambient Particles (CAPs), Standard Reference Material (SRM) 1648a, and SRM 2975 were weighed and resuspended in cell culture media supplemented according to the cell culture in use (Caceres et al., 2020). Major metallic components of the PM_2.5_ samples used in the present study are listed in Supplementary Table S1.

### 2.2 Cell culture and incubation with PM_2.5_

*THP-1-ASC-GFP cells.* Inflammasome-reporter THP-1-ASC-GFP cells (Invivogen, San Diego, CA, US) were incubated with PM_2.5_ suspensions at 1, 10, or 100 μg/mL or RPMI as control. After 6 or 24 h, inflammasome priming and ASC-specks formation were assessed by flow cytometry. Cell culture supernatants were stored at −80 °C until IL-1β detection.

*Human peripheral blood mononuclear cells (PBMCs).* Human PBMC-derived macrophages were incubated with ROFA at 100 μg/mL or RPMI for 24 h. Cell culture supernatants were stored at −80 °C until IL-1β detection.

*Bone marrow-derived macrophages (BMDMs).* Male C57BL/6J wild type, ASC-Citrine (B6.Cg-Gt(ROSA)26Sor^tm1.1(CAG−Pycard/mCitrine^*^,−CD2^*^)Dtg^/J), and NLRP3- (*Nlrp3*^*−/−*^) or Caspase-1- (*Casp1*^*−/−*^) deficient mice on a C57BL/6 background (Jackson Laboratories, Bar Harbor, ME, US) were used in accordance with local and institutional guidelines. Murine BMDMs were differentiated from tibial and femoral bone marrow aspirates and incubated with ROFA at 100 μg/mL or RPMI for 6 or 24 h. Inhibitors were added prior to ROFA stimulation and include: 3 μM MCC950 (Invivogen), 100 μg/mL anti-TNF-α antibody (clone MP6-XT22, BioLegend, San Diego, CA, US), 20 μM Cytochalasin D (Sigma-Aldrich, St. Louis City, MO, US), and 5 μM S1QEL 1.1 or S3QEL 2 (Sigma-Aldrich). Cell culture supernatants were stored at −80 °C until the evaluation of cytokine levels. Additional assays in BMDMs using PM_2.5_ samples other than ROFA are described in the Data Supplement.

### 2.3 NLRP3 inflammasome priming and activation

THP-1-ASC-GFP cells and ASC-Citrine BMDMs were acquired in a FACSCanto II equipment (BD Biosciences, Franklin Lakes, NJ, US). Gates for priming and ASC-specks formation were established according to the relative distribution of the cell population in the FITC-W and FITC-A channels (Hoss et al., 2018). Data were analyzed by FlowJo Software version 10.8.1 (Tree Star, Ashland, OR, US). In addition, ASC-Citrine BMDMs were imaged with a SP8 confocal microscope (Leica Microsystems, Wetzlar, Germany) equipped with a 63 × /1.40 oil objective. Data processing was performed with the LAS X Life Science software (Leica Microsystems).

### 2.4 Cytokine profiles

IL-1β and IL-18 were quantified by ELISA in cell culture supernatants using the Human or Mouse IL-1β/IL-1F2 Quantikine Kit (R&D Systems), or the IL-18 Mouse IL-18 DuoSet kit (R&D Systems). TNF-α, IL-6, and CCL2 levels were quantified by the CBA Mouse Inflammation Kit (BD Biosciences) in cell culture supernatants according to manufacturer's instructions.

### 2.5 Electron tomography

BMDMs were plated in 6-well plates containing 6 mm sapphire discs. Cells were fixed and processed as early described (Rog-Zielinska et al., 2016). Imaging was performed at the Electron Microscopy Core Facility of the European Molecular Biology Laboratory (EMBL) (Heidelberg, Germany). Image reconstruction and segmentation were conducted using IMOD software (Rog-Zielinska et al., 2021).

### 2.6 Lysosomal disruption

BMDMs were loaded with 1 μg/mL Acridine Orange for 20 min (Antunes et al., 2001). Images were acquired using a Leica SP8 confocal microscope (Leica Microsystems) and data processing was performed with the LAS X Life Science software (Leica Microsystems). Additional samples were acquired in a FACSCanto II equipment (BD Biosciences) and lysosomal rupture was followed by the loss of red fluorescence from the acidic lysosomal compartment in the PerCP-Cy5.5 channel. Data were analyzed by FlowJo Software (Tree Star).

### 2.7 Inhibition of K^+^ efflux

BMDMs were incubated with increasing extracellular K^+^ concentration ([K^+^]_ex_) to inhibit the electrochemical gradient that drives K^+^ efflux (Gross et al., 2016). Cell culture supernatants were stored at −80 °C until IL-1β detection.

### 2.8 Mitochondrial function assessment

Oxygen consumption rate (OCR) was evaluated using a Seahorse XF96 Extracellular Flux Analyzer (Agilent, CA, US). Respiratory chain uncoupler and inhibitors were 1 μM FCCP, 1 μM Oligomycin A, 2 μM Antimycin A, and 2 μM Rotenone. Data were analyzed using the Seahorse XF Cell Mito Stress Test Report Generator Software (Agilent). Mitochondrial O_2_^•-^ production was assessed in wild type BMDMs incubated with 5 μM MitoSOX for 20 min in the dark at 37 °C. Samples were acquired in a FACSCanto II equipment (BD Biosciences). Mitochondrial O_2_^•-^ production was followed as an increase in red fluorescence in the PE channel. Data were analyzed by FlowJo Software (Tree Star) (Caceres et al., 2020).

### 2.9 Statistics

Data are presented as mean ± SEM from at least three independent experiments. Unpaired Student's *t*-test was used to analyze the differences between two groups. One-way ANOVA followed by Dunnett's *post hoc* test or two-way ANOVA followed by uncorrected Fisher's test were performed to evaluate differences between more than two groups. Statistical significance was considered at *p* < 0.05.

## 3 Results

### 3.1 PM_2.5_ promotes inflammasome engagement and IL-1β release in human THP-1 cells and primary monocyte-derived macrophages

**Fig. 1:**
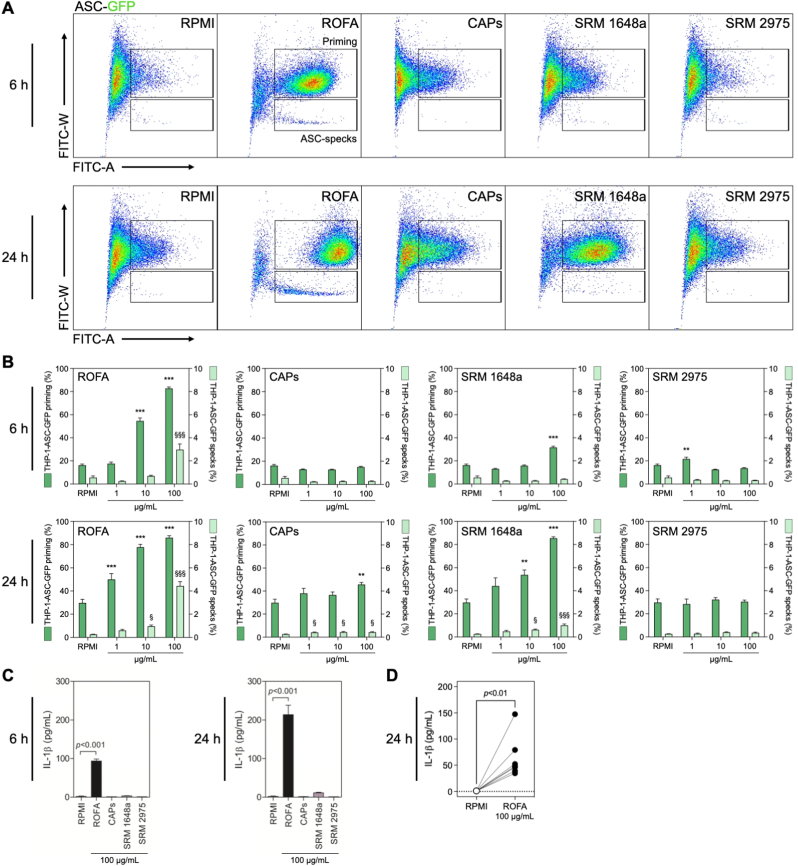
ROFA induces inflammasome priming, ASC-specks formation, and IL-1β secretion in human monocytes and macrophages. (A) Representative dot plots of inflammasome-reporter THP-1-ASC-GFP cells incubated with different PM_2.5_ surrogates at 100 μg/mL for 6 or 24 h. (B) Quantification of ASC-GFP fluorescence indicative of inflammasome priming and specks formation in THP-1-ASC-GFP cells incubated with increasing PM_2.5_ concentrations for 6 or 24 h. (C) IL-1β levels in cell culture supernatants from THP-1-ASC-GFP cells after PM_2.5_ incubation at 100 μg/mL for 6 or 24 h. (D) IL-1β release in cell culture supernatants from monocyte-derived macrophages obtained from differentiated PBMCs from healthy donors and incubated with ROFA at 100 μg/mL for 24 h. Data are presented as mean ± SEM from at least three independent experiments. *p < 0.05, **p < 0.01, and ***p < 0.001 versus RPMI (priming). ^§^*p* < 0.05 and ^§§§^*p* < 0.001 versus RPMI (specks).

Exposure to PM_2.5_ has been associated with inflammasome activation and IL-1β production (Ding et al., 2019; Du et al., 2019). Whether the chemical heterogeneity of PM_2.5_ directly affects inflammasome activation and IL-1β release, however, remains unclear (Sayan and Mossman, 2016). To test the capacity of different PM_2.5_ surrogates to induce inflammasome activation, monocytic inflammasome-reporter THP-1-ASC-GFP cells were incubated with different PM_2.5_ surrogates for 6 or 24 h at varying concentrations. Changes in GFP-fluorescence, indicative of inflammasome priming and ASC-specks formation, were quantified by flow cytometry (Fig. 1A). We detected a dose-dependent inflammasome activation after incubation with ROFA particles (Fig. 1B). While the priming signal was already significantly increased after 6 h of ROFA at 10 μg/mL, a 5-fold increase in both priming and ASC-specks formation was evident at 100 μg/mL. After 24 h, ROFA particles induced inflammasome priming and ASC-specks formation at every tested concentration with a maximum increase of 3- and 17-fold, respectively, at 100 μg/mL. In contrast, we detected only a modest response to CAPs and SRM 1648a (Fig. 1B). Because ASC-specks formation alone does not provide a definitive proof of full inflammasome activation, we quantified the levels of IL-1β in cell culture supernatants from THP-1-ASC-GFP cells exposed to the different PM_2.5_ samples at 100 μg/mL. We found a significant increase in IL-1β release following incubation with ROFA already after 6 h and more sustained after 24 h, while we observed no substantial IL-1β production for all other PM_2.5_ surrogates (Fig. 1C). To confirm that ROFA promotes inflammasome activation in human primary cells, PBMCs isolated from healthy donors and differentiated to monocyte-derived macrophages were incubated with ROFA for 24 h at 100 μg/mL. Consistent with the effect on THP-1-ASC-GFP cells, ROFA incubation led to a significant release of bioactive IL-1β (Fig. 1D). Our findings indicate that transition metal-rich ROFA particles are capable of promoting inflammasome activation and IL-1β release in myeloid cells.

### 3.2 ROFA induces both priming and activation signals for NLRP3 inflammasome-dependent IL-1β secretion in mice BMDMs

**Fig. 2:**
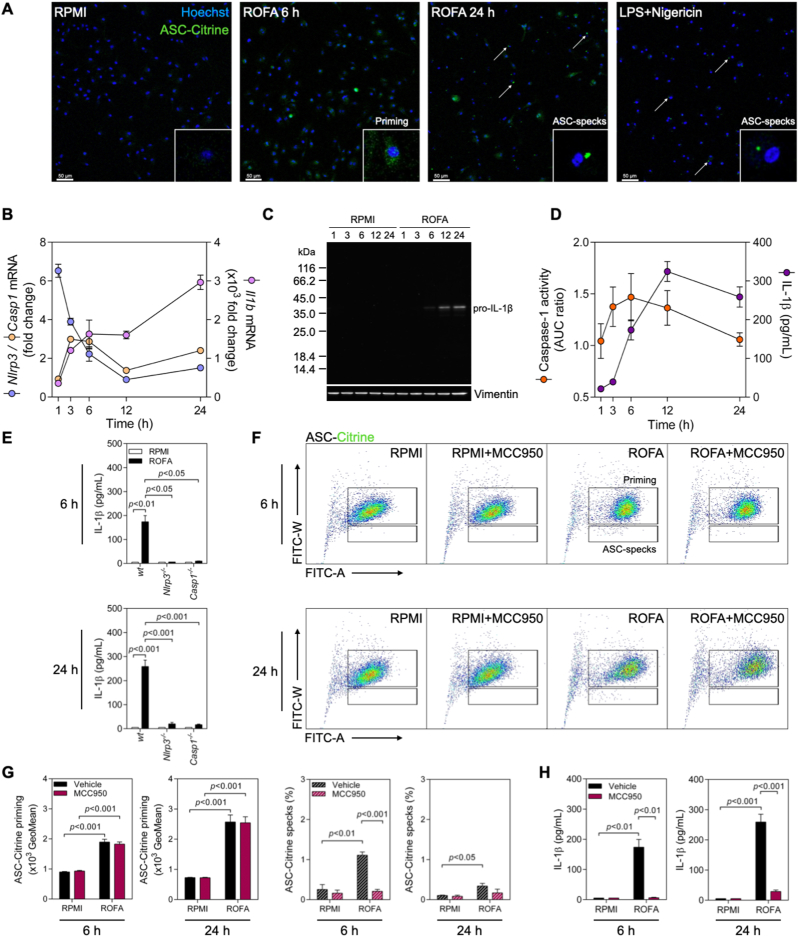
ROFA promotes the NLRP3 inflammasome-dependent release of IL-1β in murine BMDMs. (A) Confocal microscopy of BMDMs from inflammasome-reporter ASC-Citrine mice incubated with ROFA at 100 μg/mL for 6 or 24 h. White arrows indicate ASC-specks formation. LPS stimulation at 20 ng/mL for 4 h followed by the addition of 5 μM Nigericin for 2 h was used as a positive control. Time course analysis of (B) *Nlrp3*, *Casp1*, and *Il1b* mRNA expression (C) pro-IL-1β protein levels, and (D) Caspase-1 activity and IL-1β release in BMDMs from wild type (wt) mice incubated with ROFA at 100 μg/mL. (E) IL-1β release in cell culture supernatants from wt, *Nlrp3*^*−/−*^, and *Casp1*^*−/−*^ BMDMs incubated with ROFA at 100 μg/mL for 6 or 24 h. (F) Representative dot-plots of ASC-Citrine BMDMs incubated with ROFA at 100 μg/mL for 6 or 24 h, with or without pre-incubation with MCC950. (G) Quantification of ASC-Citrine fluorescence in ASC-Citrine BMDMs incubated with ROFA at 100 μg/mL for 6 or 24 h. (H) IL-1β levels in cell culture supernatants from wt BMDMs incubated with ROFA at 100 μg/mL for 6 or 24 h, with or without pre-incubation with MCC950. Data are presented as mean ± SEM from at least three independent experiments.

To establish a direct role of the NLRP3 inflammasome in IL-1β secretion, we tested BMDMs from wild type, reporter ASC-Citrine, and *Nlrp3*^*−/−*^ and *Casp1*^*−/−*^ mice on a C57BL/6 background, as well as the inhibitor of NLRP3 oligomerization, MCC950. First, as observed in THP-1-ASC-GFP cells, ROFA induced a significant release of IL-1β in wild type BMDMs after 6 and 24 h, while a negligible effect was found for the other PM_2.5_ samples tested (Supplementary Fig. S1). Similarly, IL-18 levels were also increased following ROFA incubation after 24 h (Supplementary Fig. S2). Unchanged LDH levels in BMDM supernatants are indicative of preserved cell viability in our experimental cell culture conditions (Supplementary Fig. S3). Then, we confirmed inflammasome priming and assembly following ROFA incubation in BMDMs from ASC-reporter mice by confocal microscopy (Fig. 2A).

To further clarify the dynamics of NLRP3 inflammasome priming and activation, we constructed a time course of gene transcripts involved in NLRP3 engagement and IL-1β release. Wild type BMDMs incubated with ROFA showed an early upregulation of *Nlrp3* transcripts (coding for NLRP3) by up to 6.5-fold already after 1 h, which normalized after 12 h. *Casp1* (coding for Caspase-1) demonstrated a 3-fold increase after 3 h before returning to baseline levels. *Il1b* mRNA expression (coding for IL-1β) continuously rose up to 3x10^3^-fold after 24 h (Fig. 2B). In parallel, increased pro-IL-1β protein levels in BMDM cell lysates are indicative of the upregulation of immature IL-1β following incubation with ROFA (Fig. 2C). Caspase-1 enzymatic activity followed a similar trend to that of *Casp1* dynamics, peaking at 6 h after ROFA stimulation. Accordingly, IL-1β release in BDMDs culture media increased over time, reaching a maximum after 12 h of ROFA incubation (Fig. 2D). NLRP3 and Caspase-1 involvement in IL-1β release was validated in BMDMs from *Nlrp3*^*−/−*^ and *Casp1*^*−/−*^ mice, as neither *Nlrp3*^*−/−*^ nor *Casp1*^*−/−*^ BMDMs secreted IL-1β into the culture media following ROFA incubation for 6 or 24 h (Fig. 2E).

In addition, we analyzed priming and ASC-specks formation in the presence of the NLRP3 inflammasome-specific inhibitor MCC950 by flow cytometry (Fig. 2F). ASC-Citrine BMDMs incubated with ROFA showed an increased priming signal compared to RPMI, regardless of MCC950 pre-incubation. This was expected, as MCC950 specifically blocks NLRP3 oligomerization and does not interfere with the priming process (Coll et al., 2015). On the other hand, the increase in ASC-specks formation observed after ROFA incubation was inhibited by MCC950 (Fig. 2G). In accordance with these findings, pre-incubation with MCC950 efficiently prevented IL-1β secretion after 6 and 24 h (Fig. 2H). These results confirm that IL-1β release is NLRP3 dependent in our model and suggest that ROFA participates in both inflammasome priming and activation signals.

### 3.3 The pro-inflammatory phenotype switch induced by ROFA in BMDMs is partially dependent on NLRP3 inflammasome activation

**Fig. 3:**
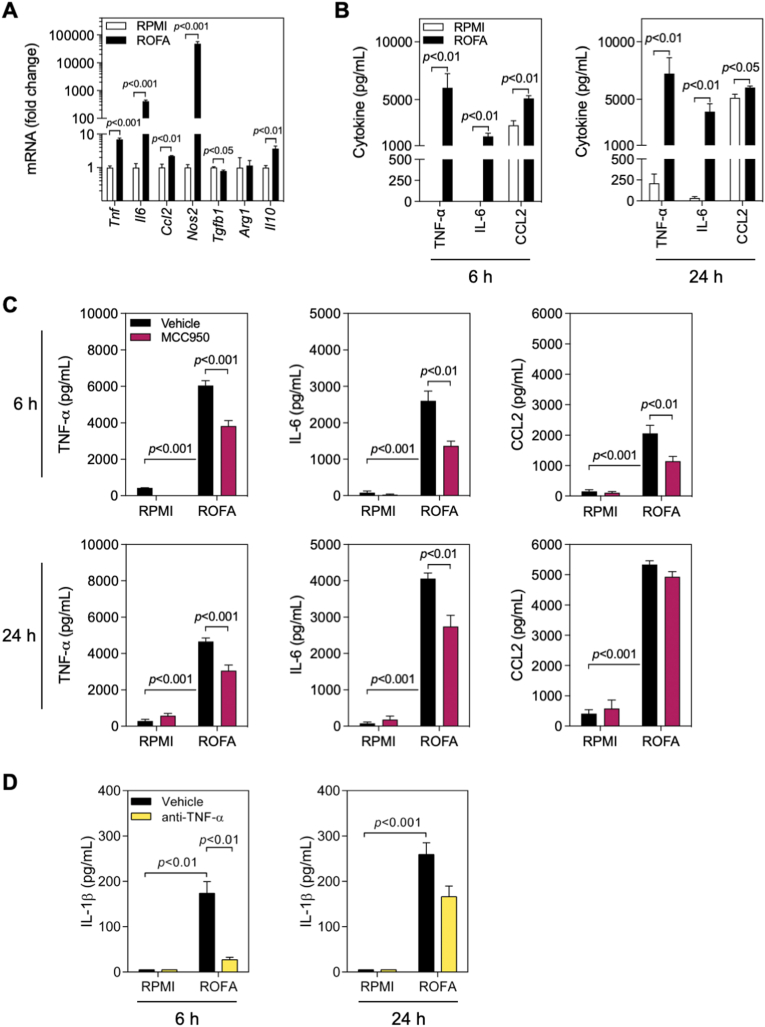
The pro-inflammatory phenotype induced by ROFA incubation in BMDMs partially depends on the NLRP3 inflammasome. (A) Gene expression in wild type BMDMs incubated with ROFA at 100 μg/mL for 24 h. (B) Cytokine levels in cell culture supernatants from BMDMs incubated with ROFA at 100 μg/mL for 6 or 24 h, and (C) with or without pre-incubation with MCC950. (D) IL-1β levels in cell culture supernatants from BMDMs incubated with ROFA at 100 μg/mL for 6 or 24 h, and with or without pre-incubation with a blocking anti-TNF-α antibody. Data are presented as mean ± SEM from at least three independent experiments.

Previous studies have shown that an exposure to PM_2.5_ induces TNF-α, IL-6, and CCL2 production in macrophages (Caceres et al., 2020; Fu et al., 2020; Marchini et al., 2016). We therefore speculated that NLRP3-dependent IL-1β release may be partially responsible for this pro-inflammatory phenotype. First, we incubated BMDMs with ROFA and quantified mRNA transcripts indicative of macrophage polarization. After 6 h, we detected increased mRNA levels coding for the pro-inflammatory cytokines TNF-α (*Tnf)* and IL-6 (*Il6)*, and of iNOS (*Nos2*), together with significantly downregulated TGF-β1 (*Tgfb1*) and Arginase-1 (*Arg1*) (Supplementary Fig. S4). At 24 h, CCL2 (*Ccl2*) was also upregulated, while genes coding for anti-inflammatory mediators were only moderately upregulated (IL-10, *Il10*), unchanged (Arginase-1, *Arg1*), or even significantly downregulated (TGF-β1, *Tgfb1*) (Fig. 3A). Consistently with inflammatory gene expression analysis, the levels of TNF-α, IL-6, and CCL2 were significantly increased 6 and 24 h after ROFA incubation (Fig. 3B). A minor effect on TNF-α and IL-6 release was also observed following incubation with SRM 1648a particles (Supplementary Fig. S5), as reported previously (Gawda et al., 2018). Taken together, these data indicate that ROFA ignites a pro-inflammatory phenotype in BMDMs, consistent with a classically activated M1-like polarization.

To test the contribution of NLRP3 inflammasome activation on the observed cytokine release, BMDMs were pre-treated with MCC950 before ROFA incubation. Although cytokine production was not completely blocked, pre-incubation with MCC950 led to a 37% decrease in TNF-α secretion, and a 47% and 44% reduction in IL-6 and CCL2 release, respectively, after stimulation with ROFA for 6 h. Similar findings were obtained after 24 h (Fig. 3C). It has been reported that TNF-α signaling leads to enhanced gene expression of NLRP3 inflammasome components, therefore acting as a priming signal (Swanson et al., 2019). Accordingly, pre-incubation with a blocking anti-TNF-α antibody significantly reduced IL-1β secretion by ROFA (Fig. 3D), suggesting that TNF-α partially contributes to NLRP3 inflammasome priming. These findings indicate that blocking NLRP3 inflammasome engagement may provide a novel therapeutic anti-inflammatory strategy, by reducing some of the inflammatory consequences of PM_2.5_ without fully neutralizing other inflammatory mediator release.

### 3.4 ROFA accumulates in vesicles inside BMDMs and induces loss of mitochondrial membrane integrity

**Fig. 4:**
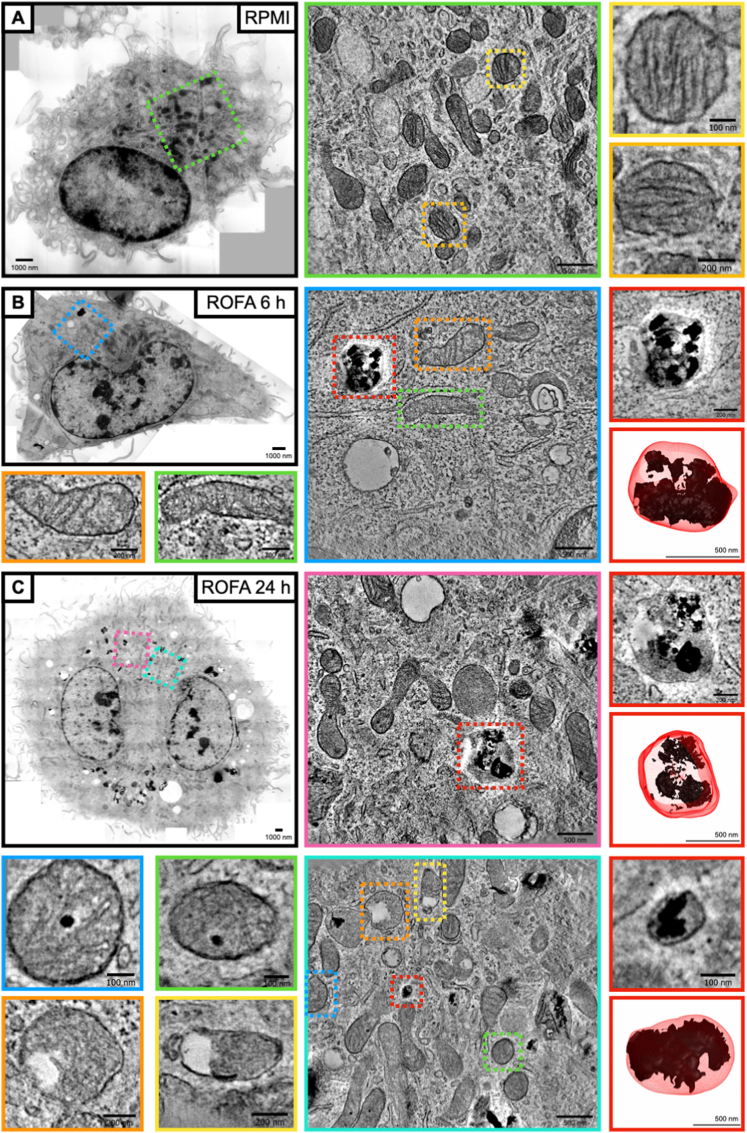
ROFA uptake by BMDMs accumulates in vesicles and reaches mitochondria leading to structural damage. Representative electron tomography overview of BMDMs after incubation with (A) RPMI for 24 h, or ROFA at 100 μg/mL for (B) 6 or (C) 24 h. (A) Control BMDMs show preserved mitochondrial integrity and absence of particle-containing vesicles (green frame close-up). Higher magnification images show preserved mitochondrial structure (yellow and orange frames). (B) BMDMs incubated with ROFA for 6 h show particle accumulation in vesicles and signs of early structural damage in mitochondria (blue frame close-up). Higher magnification images show mild mitochondrial structure alterations, i.e. local absence of cristae (orange and green frames). (C) BMDMs incubated with ROFA for 24 h show a higher density of particle-containing vesicles and severe structural alterations in mitochondria (magenta and cyan frame close-ups). Higher magnification images show ROFA inside mitochondria (blue and green frames) and prominently altered mitochondrial structure (yellow and orange frames). (B and C) Vesicles containing ROFA particles after 6 or 24 h of incubation, and 3D reconstruction modelling, are shown in red frames. (For interpretation of the references to colour in this figure legend, the reader is referred to the Web version of this article.)

We and others have previously shown that macrophages are able to readily uptake PM_2.5_ (Caceres et al., 2020; Li et al., 2003; Marchini et al., 2022). In the present study, we found that inhibition of phagocytosis with Cytochalasin D attenuated IL-1β release in BMDMs incubated with ROFA (Supplementary Fig. S6). This result highlights the need of PM_2.5_ internalization for NLRP3 activation and IL-1β secretion. However, a specific subcellular localization site for PM_2.5_ accumulation remains unclear. First, we confirmed the uptake of ROFA by cultured BMDMs using standard bright-field microscopy (Supplementary Fig. S7). Then, electron tomography imaging demonstrated the accumulation of particles inside intracellular vesicles of BMDMs after 6 or 24 h of incubation (Fig. 4A–C). 3D reconstructions based on electron tomography volumes depict enclosure of particles inside these vesicles (Fig. 4, red frames). ROFA uptake also promoted evident ultrastructural changes in mitochondria. While BMDMs incubated with ROFA for 6 h showed early signs of mitochondrial cristae disruption, mitochondrial damage became more evident after 24 h (Fig. 4B and C). Furthermore, ROFA seems to also accumulate inside mitochondria at the later time point. Taking these findings into consideration, we next addressed lysosomal leakage and mitochondrial dysfunction as possible NLRP3 inflammasome activation mechanisms triggered by ROFA.

### 3.5 Lysosomal disruption, early K^+^ efflux, and delayed O_2_^•-^ production by mitochondrial respiratory complex I, but not III, drive NLRP3 inflammasome activation by ROFA

**Fig. 5:**
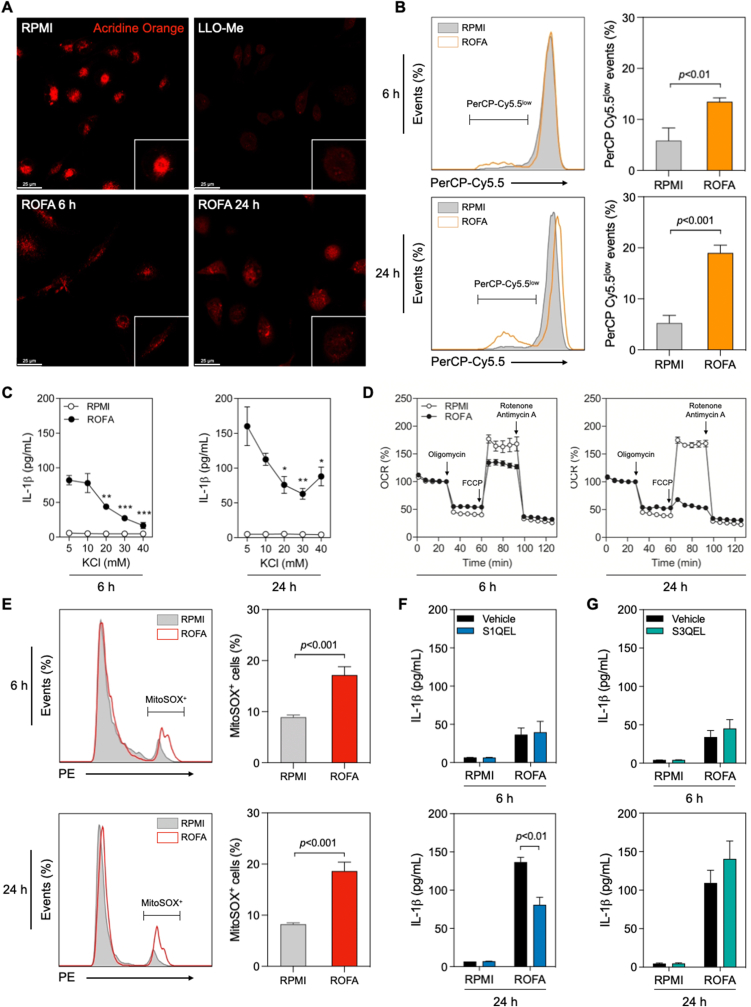
Lysosomal disruption, K^+^ efflux, and mitochondrial dysfunction drive NLRP3 activation and IL-1β release following ROFA incubation. (A) Confocal microscopy of wild type BMDMs incubated with ROFA at 100 μg/mL for 6 or 24 h were stained with Acridine Orange. LLO-Me was used as positive control for lysosomal destabilization. (B) Representative histograms for Acridine Orange staining assessed by flow cytometry in BMDMs incubated with ROFA at 100 μg/mL. Quantification of PerCP-Cy5.5^low^ events was indicative of lysosomal disruption after 6 or 24 h. (C) IL-1β levels in cell culture supernatants from BMDMs pre-incubated with increasing [K^+^]_ex_, followed by incubation with ROFA at a concentration of 100 μg/mL for 6 or 24 h. (D) Mitochondrial oxygen consumption rate (OCR) was assessed by the Seahorse MitoStress Test in BMDMs incubated with ROFA at 100 μg/mL for 6 or 24 h. (E) Representative histograms of MitoSOX staining assessed by flow cytometry in BMDMs incubated with ROFA at 100 μg/mL for 6 or 24 h. Quantification of MitoSOX^+^ cells indicated mitochondrial O_2_^•-^ production after 6 or 24 h of ROFA incubation. (F and G) IL-1β levels in cell culture supernatants from BMDMs with or without pre-incubation with (F) a selective O_2_^•-^ production inhibitor targeting mitochondrial Complex I (S1QEL 1.1) or (G) Complex III (S3QEL 2), followed by incubation with ROFA at 100 μg/mL for 6 or 24 h. Data are presented as mean ± SEM from at least three independent experiments. *p < 0.05, **p < 0.01, ***p < 0.001 versus ROFA at 5 mM [K^+^]_ex_.

NLRP3 agonists can induce multiple molecular and cellular signaling events that promote NLRP3 oligomerization and IL-1β maturation (Kelley et al., 2019). Lysosomal leakage was reported as one key mechanism leading to NLRP3 inflammasome engagement following exposure to particulate stimuli, such as silica (Jessop et al., 2017). BMDMs incubated with RPMI alone showed an intense red fluorescent signal after staining with Acridine Orange, which indicates preserved lysosomal integrity. On the contrary, BMDMs incubated with LLO-Me, a lysosomal permeabilizing agent, had a characteristically dimmer signal. After incubation with ROFA, BMDMs showed a lower signal intensity than the control (Fig. 5A), indicating that PM_2.5_ uptake promotes lysosomal disruption. Changes in Acridine Orange fluorescence were quantified by flow cytometry and showed a 2.3-fold increase in PerCP-Cy5.5^low^ events after 6 h of incubation with ROFA, while a 3.7-fold increase was observed after 24 h of incubation with ROFA (Fig. 5B).

K^+^ efflux is another established trigger of inflammasome activation and IL-1β release (Koumangoye, 2022). To test the impact of K^+^ efflux, BMDMs were incubated with increasing [K^+^]_ex_ while simultaneously exposed to ROFA. At [K^+^]_ex_ = 20 mM, IL-1β secretion was inhibited by 48% and 53% after 6 and 24 h, respectively (Fig. 5C). These results suggest that PM_2.5_ promotes IL-1β secretion by mechanisms that are at least partially dependent on K^+^ efflux.

Mitochondrial dysfunction and enhanced O_2_^•-^ production have also been suggested to contribute to inflammasome activation (Kelley et al., 2019; Liu et al., 2018). We observed impaired mitochondrial respiration in BMDMs incubated with ROFA for 6 and 24 h, an effect likely caused by diminished ATP turnover and loss of spare respiratory capacity (Fig. 5D and Supplementary Fig. S8). A lower mitochondrial respiratory rate is frequently linked to enhanced O_2_^•-^ production (Brand, 2016). While some of the other PM_2.5_ samples tested induced a certain degree of altered mitochondrial function (e.g., CAPs and SRM 1648a particles after 24 h, Supplementary Fig. S8), only ROFA led to a relevant upregulation of mitochondrial O_2_^•-^ production (Supplementary Fig. S9). In ROFA-stimulated BMDMs, we detected a 1.9-fold increase in O_2_^•-^ production after 6 h, which rose to 2.3-fold after 24 h, as indicated by MitoSOX^+^ events (Fig. 5E). A recent interest in source-specific inhibition of O_2_^•-^ production has led to the identification of *S*uppressors of site *I*_Q_ *E*lectron *L*eak (S1QELs) and *S*uppressors of site *III*_Qo_ *E*lectron *L*eak (S3QELs), which selectively inhibit mitochondrial O_2_^•-^ release from Complex I or III, respectively (Orr et al., 2013; Orr et al., 2015). To test the link between source-specific mitochondrial O_2_^•-^ production and IL-1β release, we tested these compounds on ROFA-exposed BMDMs. While we detected no effect after 6 h, treatment with S1QEL 1.1 (Fig. 5F), but not with S3QEL 2, significantly limited IL-1β release by 41% after 24 h of ROFA incubation (Fig. 5G). Taken together, these findings show that lysosomal leakage, K^+^ efflux, mitochondrial dysfunction, and O_2_^•-^ production from Complex I drive NLRP3 inflammasome activation and IL-1β release triggered by PM_2.5_ in macrophages.

## 4 Discussion

A large body of evidence has established that both acute and chronic exposures to PM_2.5_ cause a pulmonary and systemic inflammatory response that aggravate respiratory and cardiovascular diseases (Brook et al., 2010; Marchini et al., 2020; Pope et al., 2020). This response is partially thought to be mediated by an activation of the NLRP3 inflammasome and a release of IL-1β (Duan et al., 2019; Xiong et al., 2021). However, the underlying mechanisms remain unclear. The present study aimed to identify cellular pathways that are engaged by PM_2.5_ to trigger NLRP3 inflammasome activation and IL-1β release in macrophages.

Inflammasome-reporter THP-1-ASC-GFP cells were chosen as a novel screening strategy to test PM_2.5_ samples from different anthropogenic sources (Hoss et al., 2018).Cellular mechanisms were then addressed in mice BMDMs. PM_2.5_ concentrations and time points were selected according to previous reports (Caceres et al., 2020; Lasagni Vitar et al., 2018; Magnani et al., 2016; Zheng et al., 2018), and to avoid significant cytotoxicity in our experimental conditions. Furthermore, the chosen time points allowed us to differentially study the cellular mechanisms that might dominate NLRP3 inflammasome priming (first signal) or assembly (second signal) following the incubation with PM_2.5_. Our findings demonstrate that different sources of PM_2.5_ can induce priming and ASC-specks formation with a considerably smaller effect for CAPs and SRM 1648a compared to ROFA. This may be explained by the higher content of transition metals in ROFA and their ability to generate oxidants through Haber-Weiss and Fenton-like chemical reactions (Magnani et al., 2016; Pattanaik et al., 2012). Accordingly, metallic particles of transition metals present in ROFA, such as Ti and Cr, have been shown to activate the NLRP3 inflammasome in primed human primary macrophages (Jamsen et al., 2020). The increase in oxidants production upon incubation with PM_2.5_ has been linked to impaired cellular redox metabolism and the upregulation of inflammatory-associated transcription factors, including NF-κB. Ultimately, NF-κB-dependent pathways result in pro-inflammatory cytokine release (Marchini, 2023; Valacchi et al., 2020). In line with this concept, variable IL-1β production has been reported in PMA-differentiated THP-1 cells incubated with PM_2.5_ of different composition (Cao et al., 2022). A recent study showed that PM_2.5_ metal chelation reduces mitochondrial O_2_^•-^ production and IL-1β release in THP-1 cells (Zheng et al., 2022). In our study, while other particles showed some degree of inflammasome activation and pro-inflammatory cytokine release, ROFA was the PM_2.5_ sample with the strongest biological activity and was, therefore, chosen for subsequent mechanistic assays.

The effects described here were consistent across different origins of BMDMs in wild type, transgenic, and inflammasome-deficient mice. Using ASC-Citrine reporter BMDMs, we were able to confirm that PM_2.5_ incubation induces inflammasome priming and ASC-specks formation. While the ASC adaptor protein is shared by many types of inflammasomes (i.g. AIM2, IFI16, NLRP1, NLRP3, NLRP6, and NLRP7), the NLRP3 inflammasome is best characterized and has been mechanistically linked to several inflammatory pathologies (Mangan et al., 2018). Rapid upregulation of *Nlrp3*, *Casp1*, and *Il1b* transcriptomes highlights the ability of ROFA to aid in the priming of the NLRP3 inflammasome in macrophages (Ren et al., 2022). Consistently, pro-IL-1β protein levels and Caspase-1 enzymatic activity were increased after incubation with ROFA. IL-1β release to cell culture supernatants indicates that ROFA particles are also able to promote a functional assembly of the NLRP3 inflammasome. Other members of the inflammasome family have also been reported to participate in IL-1β release after stimulation with environmental stressors, such as NLRP1 in O_3_-exposed human primary keratinocytes and HaCaT cells (Ferrara et al., 2022). In our study, we found that IL-1β secretion was completely abolished in *Nlrp3*^*−/−*^ and *Casp1*^−/−^ BMDMs, which strongly suggests that PM_2.5_ incubation leads to NLRP3 inflammasome-dependent Caspase-1 cleavage of pro-IL-1β and IL-1β release by macrophages.

A specific inhibition of the NLRP3 inflammasome has been discussed as a promising therapeutic approach to modulate IL-1β secretion in the context of inflammatory diseases (Olsen et al., 2022). MCC950 is a small molecule specifically targeting the NLRP3 NACHT domain, thereby blocking NLRP3 oligomerization and inflammasome function (Coll et al., 2019). MCC950 has effectively reduced atherosclerotic lesion development and infarct size in animal models (van der Heijden et al., 2017; van Hout et al., 2017) and limited pyroptosis in PMA-differentiated THP-1 cells (Jia et al., 2021). Our results show that MCC950 reduced IL-1β secretion in ROFA-exposed BMDMs by preventing ASC-specks formation. Previous reports have established that PM_2.5_ induces a pro-inflammatory phenotype in macrophages (Caceres et al., 2020; Zhao et al., 2016). In the present study, analysis of inflammatory gene expression and cytokine release indicates a ROFA-induced shift that is consistent with a classically activated M1-like polarization. At the evaluated time points, this response was partially dependant on IL-1β, which highlights the complex interplay of the inflammatory pathways regulated by PM_2.5_. Furthermore, we showed that PM_2.5_-induced TNF-α secretion contributes as a priming signal for the NLRP3 inflammasome. In line with this finding, we have previously reported that TNF-α mediates systemic effects of PM_2.5_ exposure, such as the upregulation of endothelial adhesion molecules (ICAM-1 and VCAM-1) and CD11b activation in myeloid cells (Marchini et al., 2016). Moreover, impaired cardiac mitochondrial and contractile function in PM_2.5_-exposed mice was partially restored by pre-treatment with a chimeric anti-TNF-α monoclonal antibody (Infliximab) (Marchini et al., 2015). While the pharmacological modulation of the NF-κB pathway can exacerbate NLRP3-dependent inflammation (Afonina et al., 2017), MCC950 has effectively reduced plasma TNF-α, IL-6, and IL-1β levels in PM_2.5_-exposed mice (Zeng et al., 2021). In the present study, we confirmed that IL-1β is one of the key regulators of downstream pro-inflammatory cytokine production triggered by PM_2.5_ in macrophages. Accordingly, IL-1β blockade did not entirely blunt inflammation, as shown by residual pro-inflammatory cytokine release in the presence of MCC950 after ROFA incubation.

**Fig. 6:**
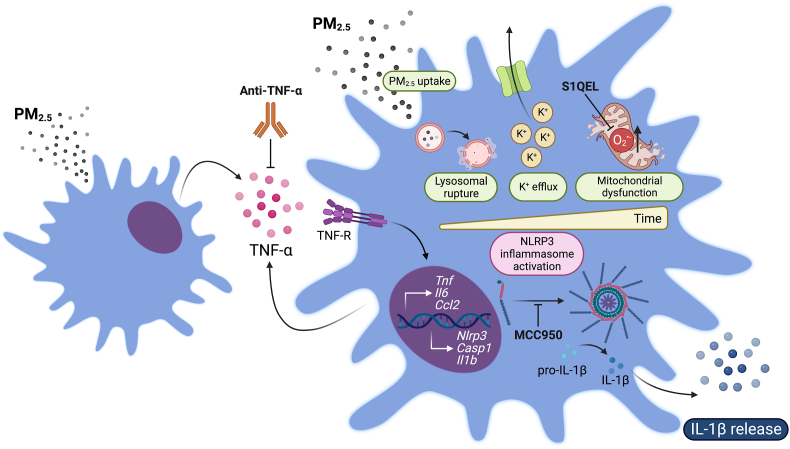
Mechanistic insights into NLRP3 inflammasome activation and IL-1β release by PM_2.5_. PM_2.5_ uptake by macrophages promotes gene expression of pro-inflammatory cytokines and inflammasome priming (i.e. upregulation of *Nlrp3*, *Casp1*, and *Il1b*). Lysosomal rupture, K^+^ efflux, and O_2_^•-^ production from mitochondrial Complex I sequentially promote NLRP3 inflammasome oligomerization, ASC-specks formation, pro-IL-1β cleavage by activated Caspase-1, and IL-1β release. PM_2.5_-induced inflammation involves cytokine production, such as of TNF-α, which further contributes to NLRP3 inflammasome activation and IL-1β production. TNF-α blockade, O_2_^•-^ scavenging by S1QEL 1.1, and inhibition of NLRP3 oligomerization by MCC950 reduces PM_2.5_-induced IL-1β release in macrophages.

Various intracellular signals have been proposed for the induction of the second step of NLRP3 inflammasome activation (Hoseini et al., 2018). In this study, we describe an integration of cellular pathways that lead to NLRP3 inflammasome activation (summarized in Fig. 6). These include: (a) lysosomal disruption, (b) K^+^ efflux, and (c) mitochondrial dysfunction and enhanced O_2_^•-^ production. First, lysosome rupture is expected to rapidly and steadily occur following PM_2.5_ uptake, as already described for other particulate stimuli such as cholesterol crystals and silica, most likely as a consequence of continuous particle internalization (Chen et al., 2019; Hornung et al., 2008; Ural et al., 2022). Accordingly, lysosomal disruption seems to aggravate over time in our model. Down this line, we found ROFA in vesicles inside BMDMs, at both evaluated time points. Cathepsins release resulting from lysosomal membrane permeabilization have been implicated in NLRP3 oligomerization and specks formation (Campden and Zhang, 2019). Their role in NLRP3 activation by PM_2.5_ needs to be addressed in future studies considering their overlapping functions. Second, as an already defined common pathway for many NLRP3 activators (Munoz-Planillo et al., 2013), and in accordance with a previous report using THP-1 cells (Zheng et al., 2018), we found that K^+^ efflux has the potential to modulate PM_2.5_-induced IL-1β release. While IL-1β release was completely blocked at high K^+^ concentrations after 6 h of incubation with PM_2.5_, it was only partially inhibited at the 24-h time point. This may indicate that alternative routes towards IL-1β secretion may take place as a result of a longer incubation with PM_2.5_. The role of mitochondria in NLRP3 activation has been established as an effect of O_2_^•-^ production, mitophagy regulation, colocalization of mitochondria and the endoplasmic reticulum, and modulation of ion fluxes (Zhou et al., 2011). We found ROFA inside mitochondria, as well as signs of structural damage that could compromise organelle function. In line with this finding, extracellular flux analysis showed OCR alterations that intensified over time. We and others have identified NADPH oxidase 2 (NOX2) and impaired mitochondrial respiration as the main sources of enhanced O_2_^•-^ production after PM_2.5_ exposure (Caceres et al., 2020; Cui et al., 2020; Kampfrath et al., 2011; Magnani et al., 2013). It has been also shown that PM_2.5_-induced O_2_^•-^ generation from mitochondrial Complex III is required for opening calcium release-activated channels (CRAC) and IL-6 release (Soberanes et al., 2019). However, the submitochondrial O_2_^•-^ production site that is relevant for NLRP3 inflammasome engagement and IL-1β release by PM_2.5_ has not yet been characterized. By using selective inhibitors of O_2_^•-^ production from Complex I (S1QEL 1.1) or III (S3QEL 2), we show for the first time that enhanced O_2_^•-^ generation at mitochondrial respiratory Complex I, but not from Complex III, drives IL-1β release in macrophages following PM_2.5_ uptake. The fact that the most evident reduction in IL-1β levels by S1QEL 1.1 was only achieved after 24 h – when high K^+^ did not completely blocked IL-1β release and mitochondrial respiration was clearly affected – indicates that K^+^ efflux initially drives IL-1β release by PM_2.5_, yet enhanced O_2_^•-^ generation from mitochondrial respiratory Complex I dominates IL-1β production in the long term.

## 5 Conclusion

In the present study, we have characterized molecular effectors and pathways steering IL-1β release and subsequent inflammation following the uptake of air pollution PM_2.5_ by macrophages. We have identified several cellular mechanisms as potential targets for developing highly specific therapeutic approaches, including an interference with NLRP3 oligomerization by MCC950 or scavenging O_2_^•-^ production from mitochondrial respiratory Complex I. Ultimately, our findings shed light on the innate immune response that follows PM_2.5_ exposure.

## Funding

This work has received funding from the European Research Council (ERC) under the European Union's Horizon 2020 research and innovation program (grant agreement #853425). TMar, DW, OGr, ERZ, IH, and LSM are members of the SFB1425 (project #422681845), funded by the German Research Foundation (DFG). TMar was supported by funding from the Research Commission of the Faculty of Medicine of the University of Freiburg (grant agreement MAR2215-22). LC was funded by a fellowship from the German Academic Exchange Service (DAAD) and from the University of Buenos Aires. JM and PS received 10.13039/501100001659DFG funding (project #467077284). OGr received funding from SFB1160 (project #256073931), SFB/TRR167 (project #259373024), SFB1479 (project #441891347), GRK2606 (project #423813989), as well as by the ERC Starting Grant (#337689), Proof-of-Concept Grant (#966687), the EU-H2020-MSCA-COFUND EURIdoc Programme (#101034170), and from Germany's Excellence Strategy CIBSS–EXC-2189 (#390939984).

## Author statement

**Lourdes Caceres:** Conceptualization, Methodology, Investigation, Formal analysis, Writing - Original Draft, Writing - Review & Editing, Visualization, Validation; **Tijani Abogunloko:** Investigation, Formal analysis; **Sara Malchow:** Investigation, Formal analysis; **Fabienne Ehret:** Investigation, Formal analysis; **Julian Merz:** Conceptualization, Resources; **Xiaowei Li:** Investigation, Formal analysis; **Lucia Sol Mitre:** Investigation, Formal analysis; **Natalia Magnani:** Conceptualization; **Deborah Tasat:** Resources; **Timothy Mwinyella:** Investigation, Formal analysis; **Lisa Spiga:** Investigation, Formal analysis; **Dymphie Suchanek:** Investigation, Formal analysis; **Larisa Fischer:** Investigation, Formal analysis; **Oliver Gorka:** Formal analysis, Writing - Review & Editing; **Mark Colin Gissler:** Resources; **Ingo Hilgendorf:** Resources; **Peter Stachon:** Resources; **Eva Rog-Zielinska:** Investigation, Formal analysis, Writing - Review & Editing; **Olaf Groß:** Resources, Funding acquisition; **Dirk Westermann:** Resources; **Pablo Evelson:** Conceptualization, Supervision; **Dennis Wolf:** Conceptualization, Resources, Supervision, Project administration, Writing - Review & Editing, Funding acquisition; **Timoteo Marchini:** Conceptualization, Methodology, Investigation, Formal analysis, Validation, Writing - Original Draft, Writing - Review & Editing, Visualization, Supervision, Project administration, Funding acquisition.

## Declaration of competing interest

The authors declare that they have no known competing financial interests or personal relationships that could have appeared to influence the work reported in this paper.

## Data Availability

Data will be made available on request.
