# Supplementary material for: Molecular mechanisms underlying NLRP3 inflammasome activation and IL-1β production in air pollution fine particulate matter (PM2.5)-primed macrophages
Source: Environ Pollut. 2024 Jan 15;341:122997. doi: 10.1016/j.envpol.2023.122997 (PMC10804998; doi:10.1016/j.envpol.2023.122997)
Supplement: Multimedia component 1 [file mmc1.pdf]

Data Supplement

Molecular mechanisms underlying NLRP3 inflammasome activation and IL-1β production in air pollution fine particulate matter ($PM_{2.5}$)-primed macrophages

Lourdes Caceres, Tijani Abogunloko, Sara Malchow, Julian Merz, Xiaowei Li, Fabienne Ehret, Lucia Sol Mitre, Natalia Magnani, Deborah Tasat, Timothy Mwinyella, Lisa Spiga, Dymphie Suchanek, Larissa Fischer, Oliver Gorka, Mark Colin Gissler, Ingo Hilgendorf, Peter Stachon, Eva Rog-Zielinska, Olaf Groß, Dirk Westermann, Pablo Evelson, Dennis Wolf, Timoteo Marchini

Supplementary Materials and Methods

1. $PM_{2.5}$ sample collection and preparation

All $PM_{2.5}$ samples were weighed and resuspended in cell culture media supplemented according to the cell culture in use, to a final concentration of 100 µg/mL. Additionally, 10 and 1 µg/mL dilutions were obtained from the original suspension. Major metallic components of the $PM_{2.5}$ samples used in the present study are listed in Supplementary Table S1.

1.1. Residual Oil Fly Ash (ROFA)

ROFA particles were collected as a by-product of fossil fuel combustion for power generation at Boston Edison Co., Mystic Power Plant (Mystic, CT, US) during burning of low-sulphur fuel oil N° 6 and were kindly provided by Dr. Godleski (Harvard School of Public Health, Boston, MA, US). ROFA samples used in the present work have an average aerodynamic diameter of 2.1 ± 1.6 µm and a high content of transition metals, namely Fe (III), Ni (II), and V (IV) as water soluble sulphates (Caceres et al., 2020).

1.2. Concentrated Ambient Particles (CAPs)

CAPs samples were collected using a Harvard Ambient Particle Concentrator (HAPC) located at the Harvard School of Public Health and were kindly provided by Dr. González-Flecha. CAPs size ranges from 0.1 to 2.5 µm and their elemental composition is predominantly defined by S and Si, with low transition metal content (Magnani et al., 2016).

1.3. Standard Reference Material (SRM) 1648a

SRM 1648a samples were collected from St. Louis City (MO, US) urban air and provided by the National Institute of Standards and Technology (NIST, Gaithersburg, MD, US). Morphological analysis indicates a mean particle diameter of 5.85 µm and elemental composition lists Fe as the most abundant transition metal. In addition, SRM 1648a has a high content of polychlorinated biphenyls (PCBs) and polycyclic aromatic hydrocarbons (PAHs) (SRM 1648a Certificate of Analysis).

1.4. SRM 2975

SRM 2975 samples are Diesel Exhaust Particles (DEP) collected from the exhaust of an industrial forklift from the M.E. Wright Donaldson Company Inc. (Minneapolis, MN, US) and provided by the NIST. Morphological and chemical composition analysis shows an average size of 1.62 ± 0.01 µm, and a high PAHs and nitro-PAHs content (SRM 2975 Certificate of Analysis). Elemental composition has been reported by (Farahani et al., 2021).

2. *Cell culture*

2.1. THP-1-ASC-GFP cell culture conditions and incubation with $PM_{2.5}$

Inflammasome-reporter THP-1-ASC-GFP cells (Invivogen, San Diego, CA, US) were cultured in RPMI 1640 supplemented with 0.29 mg/mL L-glutamine, 25 mM HEPES, 2.2 g/L $NaHCO_3$, 10% FBS, 100 U/mL penicillin, 100 µg/mL streptomycin, and 100 µg/mL normocin. THP-1-ASC-GFP cells were kept in a 5% CO_2 atmosphere and at 37 °C in a humidified incubator, and cell culture media was replaced according to manufacturer's instructions. On the day of the assay, cells were centrifuged at 500 g for 5 min at 4 °C, and the pellet was resuspended in RPMI 1640 supplemented with 0.29 mg/mL L-glutamine, 25 mM HEPES, 2.2 g/L $NaHCO_3$, 10% FBS, 100 U/mL penicillin, and 100 µg/mL streptomycin. Afterwards, THP-1-ASC-GFP cells were plated in 96-well plates at a density of $1x10^5$ cells/well and incubated with $PM_{2.5}$ suspensions at 1, 10, or 100 µg/mL. THP-1-ASC-GFP cells incubated with RPMI were used as control. After 6 or 24 hours, inflammasome priming and ASC-specks formation were assessed by flow cytometry (Hoss et al., 2018). Cell culture supernatants were stored at -80 °C until IL-1β detection by ELISA.

2.2. Human peripheral blood mononuclear cells (PBMCs) isolation, differentiation, and incubation with ROFA

Venous blood samples from 7 healthy donors (3 males and 4 females) were collected in BD Vacutainer CPT tubes and centrifuged at 1,800 g for 15 min at room temperature (22˚C). Isolated PBMCs were transferred to a T25 flask, cultured at 37 °C and 5% CO_2 in a humidified incubator, and differentiated in RPMI supplemented with recombinant human M-CSF (Immunotools GmbH, Friesoythe, Germany) at 100 ng/mL. After 6 days, monocyte-derived macrophages were plated in 96-well plates at a density of $1x10^5$ cells/well and incubated with ROFA at 100 µg/mL or RPMI for 24 hours. Cell culture supernatants were stored at -80 °C until IL-1β detection by ELISA.

2.3. Bone marrow-derived macrophages (BMDMs) preparation and incubation with $PM_{2.5}$

Male C57BL/6J wild type, ASC-Citrine (B6.Cg-Gt(ROSA)26Sor$^{tm1.1(CAG-Pycard/mCitrine*,-CD2*)Dtg}$/J), and NLRP3- (*Nlrp3*$^{-/-}$) and Caspase-1- (*Casp1*$^{-/-}$) deficient mice on a C57BL/6 background (Jackson Laboratories, Bar Harbor, ME, US) were used in accordance with local and institutional guidelines. Mice were euthanized by CO_2 inhalation and cervical dislocation. Murine BMDMs were differentiated from tibial and femoral bone marrow aspirates as previously described (Schneider et al., 2013) using recombinant human M-CSF (Immunotools) at 100 ng/mL. Cells were cultured at 37 °C and 5% CO_2 in a humidified incubator and differentiated for 6 days. Unless otherwise indicated, BMDMs were plated in 96-well plates at a density of $1x10^5$ cells/well and incubated with $PM_{2.5}$ suspensions at 100 µg/mL or RPMI for 6 or 24 hours. Sample preparation for flow cytometry included BMDMs dissociation by incubation with 0.5 mM EDTA for 5 min at 37 °C. Detached cells were then washed and resuspended in cell culture media.

2.3.1. Inhibitory approaches

The specific NLRP3 inflammasome inhibitor MCC950 (Invivogen) was used at 3 µM, 1 hour prior to ROFA stimulation. An anti-TNF-α antibody (clone MP6-XT22, BioLegend, San Diego, CA, US) was used at 100 µg/mL, 1 hour prior to ROFA stimulation. The phagocytosis and micropinocytosis inhibitor Cytochalasin D was used at 20 µM, 2 hours before ROFA stimulation. Inhibitors of superoxide anion ($O_2^{\bullet-}$) production from mitochondrial respiratory Complex I (S1QEL 1.1) or III (S3QEL 2) (Sigma-Aldrich, St. Louis City, MO, US) were used at 5 µM, 1 hour prior to ROFA stimulation.

2.3.2. Cell viability

Cell viability was assessed by the CytoTox 96 assay (Promega, Madison, WI, US), which quantitatively measures lactate dehydrogenase (LDH) released in cell culture supernatants upon cytoplasmic membrane destabilization and cell lysis.

3. *NLRP3 inflammasome priming and activation*

3.1. Flow cytometry

THP-1-ASC-GFP cells were acquired in a FACSCanto II equipment (BD Biosciences, Franklin Lakes, NJ, US) in the FL-1 channel. Gates for priming and specks formation were established according to THP-1-ASC-GFP relative distribution in the FITC-W and FITC-A channels. THP-1-ASC-GFP cells undergoing NLRP3 priming show enhanced green cytoplasmic fluorescence, thus a high FITC-A signal. Cells in the second stage of inflammasome activation display focalized fluorescence due to ASC-specks formation, thus showing a low FITC-W signal (Hoss et al., 2018). The same gating strategy was used when acquiring ASC-Citrine BMDMs. Data were analyzed by FlowJo Software version 10.8.1 (Tree Star, Ashland, OR, US).

3.2. Fluorescence microscopy

ASC-Citrine BMDMs were plated in 8-well chamber slides (Ibidi GmbH, Gräfelfing, Germany) at $5x10^4$ cells/well and left overnight until achieving full cell adherence. Next, cells were incubated with ROFA at 100 µg/mL or RPMI for 6 or 24 hours. As a positive control for ASC-specks formation, ASC-Citrine BMDMs were incubated with 20 ng/mL LPS for 4 hours followed by stimulation with 5 µM nigericin (Invivogen) for 2 hours. Cell nuclei were counterstained with 1 µg/mL Hoechst dye. Images were acquired with a SP8 confocal microscope (Leica Microsystems, Wetzlar, Germany) equipped with a 63×/1.40 oil objective. Bright field images were also acquired to evaluate BMDMs $PM_{2.5}$ uptake. Data processing was performed with the LAS X Life Science software (Leica Microsystems).

4. *qRT-PCR*

To perform a time course analysis of NLRP3-related gene expression, wild type BMDMs were plated in 12-well plates at $5x10^5$ cells/well and incubated with ROFA at 100 µg/mL or RPMI for 1, 3, 6, 12, and 24 hours. Additionally, samples from the 6- and 24-hour time point were used to build an inflammatory gene expression profile for $PM_{2.5}$-exposed BMDMs. Total RNA was purified with the RNeasy Mini Kit (Qiagen, Düsseldorf, Germany) according to manufacturer's instructions. The High-Capacity cDNA Reverse Transcription Kit (Applied Biosystems, ThermoFisher Scientific, Waltham, MA, US) was used to obtain cDNA from 200 ng of total RNA. Targets of interest were amplified using Taq-Man probes (Supplementary Table 2) and a CFX96 Touch Real-Time PCR System (Bio-Rad Laboratories, Hercules, CA, USA). Target gene expression levels were quantified by the $2^{-\Delta\Delta CT}$ method with β-actin as the housekeeping gene.

5. *IL-1β and IL-18 detection*

IL-1β was quantified by ELISA in cell culture supernatants from THP-1-ASC-GFP cells and human monocyte-differentiated macrophages using the Human IL-1β/IL-1F2 Quantikine ELISA Kit (R&D Systems) according to manufacturer's instructions. Cell culture supernatants from wild type, *Nlrp3*$^{-/-}$, and *Casp1*$^{-/-}$ BMDMs were also analyzed for IL-1β content by ELISA using the Mouse IL-1β/IL-1F2 Quantikine ELISA Kit (R&D Systems) following manufacturer's recommendations. IL-18 was quantified by ELISA in cell culture supernatants from wild type BMDMs using the Mouse IL-18 DuoSet kit (R&D Systems) following manufacturer's recommendations.

6. *Immunoblotting*

Protein levels of pro-IL-1β were detected by immunoblotting in cell lysates of BMDMs incubated with RPMI or ROFA at 100 µg/mL for 1, 3, 6, 12, or 24 hours. Cell lysates were prepared in a sample buffer containing 62.5 mM Tris–HCl (pH 6.8), 2% w/v SDS, 0.01% w/v phenol red, and 10% w/v glycerol. After heating the samples to 95 °C for 10 min, proteins were separated by SDS-PAGE (12%), 100 V for 1.5 hours at room temperature (RT). Following the transfer to nitrocellulose membranes (100 V, 100 min, 4 °C), samples were blocked with 2% skim milk powder in PBS for 1 hour at RT. Membranes were incubated overnight at 4 °C with primary antibodies (IL-1β/IL-1F2

goat pAb, #AF-401-NA, R&D Systems; Vimentin (D21H3) XP® Rabbit mAb, #5741, Cell Signaling Technology Inc., Danvers, MA, US). Corresponding secondary HRP-conjugated antibody solutions were added to the membranes for 1 hour at RT, followed by the enhanced chemiluminescence (ECL) reagent (SuperSignal West Femto Maximum Sensitivity Substrate, ThermoFisher Scientific). An ECL ChemoStar Imager (Intas Science Imaging Instruments GmbH, Göttingen, Germany) was used for detection of enhanced chemiluminescence.

7. Caspase-1 enzymatic activity

The Caspase-Glo 1 Inflammasome Assay Kit (Promega) was used for Caspase-1 activity assessment according to manufacturer's instructions. Wild type BMDMs were plated in 96-well plates at 1×10^5 cells/well and incubated with ROFA at 100 µg/mL or RPMI for 1, 3, 6, 12, and 24 hours. Cell culture supernatants were stored at -80°C until IL-1β detection by ELISA. Afterwards, cells were transferred to a white opaque plate and Caspase-1 activity-related chemiluminescent signal was followed for 60 min in an Infinite 200 PRO microplate reader (Tecan, Männedorf, Switzerland). Data were analyzed as the ratio of the area under the curve (AUC) between RPMI and ROFA samples for each evaluated time point.

8. Cytokine detection

Cytokine levels were quantified by the CBA Mouse Inflammation Kit (BD Biosciences) in cell culture supernatants from wild type BMDMs according to manufacturer's instructions.

9. Electron tomography

BMDMs were plated in 6-well plates containing 6 mm sapphire discs at 5×10^6 cell/well and left overnight to achieve full cell adherence. Cells were incubated with ROFA at 100 µg/mL or RPMI for 6 or 24 hours. Samples were chemically fixed using isosmotic Karnovsky's fixative (2.4% sodium cacodylate, 0.75% paraformaldehyde, 0.75% glutaraldehyde), processed to Epon-Araldite resin, and imaged by dual-axis electron tomography as previously described (Rog-Zielinska et al., 2016). Imaging was performed at the Electron Microscopy Core Facility of the European Molecular Biology Laboratory (EMBL) (Heidelberg, Germany). Image reconstruction and segmentation were conducted using IMOD software (Rog-Zielinska et al., 2021).

10. Lysosomal disruption

10.1. Fluorescence microscopy

Wild type BMDMs were plated in 8-well chamber slides (Ibidi) at 5×10^4 cells/well and left overnight to achieve full cell adherence. Cells were incubated with ROFA at 100 µg/mL or RPMI for 6 or 24 hours and loaded with 1 µg/mL Acridine Orange for 20 min (Antunes et al., 2001). Maximum lysosomal rupture was achieved in wild type BMDMs incubated with 1.25 mM L-leucyl-L-leucine O-methyl ester (LLO-Me) for 10 min. Images were acquired with a Leica SP8 confocal microscope (Leica Microsystems) and data processing was performed with the LAS X Life Science software (Leica Microsystems).

10.2. Flow cytometry

After 6 or 24 hours of incubation with ROFA at 100 µg/mL or RPMI, wild type BMDMs were stained with 1 µM Acridine Orange for 20 min in the dark at 37 °C, and samples were acquired in a FACSCanto II equipment (BD Biosciences). Lysosomal rupture was followed by the loss of red fluorescence from the acidic lysosomal compartment in the PerCP-Cy5.5 channel. Data were analyzed by FlowJo Software (Tree Star).

11. Inhibition of K^+ efflux

Wild type BMDMs were plated in 96-well plates at $1x10^5$ cells/well and incubated with increasing extracellular K^+ concentration ($[K^+]_{ex}$) in order to inhibit the electrochemical gradient that drives K^+ efflux (Gross et al., 2016). RPMI containing physiological $[K^+]_{ex}$ of 5 mM was used as a basal condition. In addition, RPMI was supplemented with 10, 20, 30, or 40 mM KCl to block K^+ efflux. After 6 or 24 hours of incubation with ROFA at 100 µg/mL or RPMI, cell culture supernatants were stored at -80 °C until IL-1β assessment by ELISA.

12. Mitochondrial function assessment

12.1. Mitochondrial respiration

Oxygen consumption rate (OCR) was measured using a Seahorse XF96 Extracellular Flux Analyzer (Agilent, CA, US). BMDMs were seeded in 96-well plates at $8x10^4$ cells/well in quadruplicates 6 hours before incubation with $PM_{2.5}$ suspensions at 100 µg/mL or RPMI. After 6 or 24 hours, cell culture media was switched to bicarbonate- and phenol red-free RPMI (Gibco) containing 100 ng/mL recombinant human M-CSF, 10 mM glucose, and 2 mM glutamine, and incubated for 1 hour at 37 °C in the absence of CO_2. Four baseline measurements and four response rates were measured, and the average of these rates was used for data analysis. Respiratory chain uncoupler and inhibitors were 1 µM FCCP, 1 µM Oligomycin A, 2 µM Antimycin A, and 2 µM Rotenone. OCR and indicators of mitochondrial bioenergetics were obtained using the Seahorse XF Cell Mito Stress Test Report Generator Software (Agilent) (Brand and Nicholls, 2011). Briefly, the Spare Respiratory Capacity indicates the capability of the cell to respond to an increased energetic demand, as well as how closely it is respiring at its theoretical maximum. The Coupling Efficiency is calculated from the change in basal OCR after Oligomycin addition and indicates the fraction of basal mitochondrial oxygen consumption used for ATP synthesis. The Proton Leak is the remaining basal OCR that is not coupled to ATP production, which may indicate damage to the inner mitochondrial membrane or be used as a mechanism to regulate ATP production. The Maximal Respiration represents the maximal OCR attained by adding FCCP, indicating the maximum rate of respiration that the cell can achieve. ATP Production indicates the ATP produced by the mitochondria that contributes to meeting the energetic needs of the cell. The decrease in OCR upon injection of Oligomycin represents the portion of basal respiration that was being used to drive ATP production.

12.2. Mitochondrial $O_2^{\bullet-}$ production

After 6 or 24 hours of incubation with $PM_{2.5}$ suspensions at 100 µg/mL or RPMI, wild type BMDMs were incubated with 5 µM MitoSOX for 20 min in the dark at 37 °C. Samples were acquired in a FACSCanto II equipment (BD Biosciences). Mitochondrial $O_2^{\bullet-}$ production was assessed as an increase in red fluorescence in the PE channel. Data were analyzed by FlowJo Software (Tree Star).

13. Statistics

Data are presented as mean ± SEM from at least three independent experiments. Unpaired Student's *t*-test was used to analyze the differences between two groups. One-way ANOVA followed by Dunnett's *post hoc* test or two-way ANOVA followed by uncorrected Fisher's test were performed to evaluate differences between more than two groups. Statistical significance was considered at $p<0.05$.

Supplementary Table 1. Mass fraction values of chemical elements in $PM_{2.5}$ samples[†].

Element	ROFA	CAPs	SRM 1648a
		% w/w	
Aluminium (Al)	1.90	0.37	3.43
Antimony (Sb)			~ 0.00
Arsenic (As)			0.01
Bromine (Br)			0.05
Cadmium (Cd)			0.01
Calcium (Ca)	10.23	0.34	5.84
Carbon (C)		44.89	
Cerium (Ce)			0.01
Chlorine (Cl)			0.45
Cobalt (Co)			~ 0.00
Chromium (Cr)	0.81		0.04
Copper (Cu)			0.06
Iron (Fe)	6.13	0.23	3.92
Lead (Pb)			0.66
Magnesium (Mg)		0.15	0.81
Manganese (Mn)			0.08
Mercury (Hg)			~ 0.00
Nickel (Ni)	2.43		0.01
Oxygen (O)	55.03	38.40	
Phosphorus (P)	0.46	0.20	
Potassium (K)		0.11	1.06
Rubidium (Rb)			0.01
Silicon (Si)	2.09	14.75	
Sodium (Na)	2.32		0.42
Strontium (Sr)			0.02
Sulphur (S)	11.33	0.55	5.51
Titanium (Ti)	0.21		0.40
Vanadium (V)	6.44		0.01
Zinc (Zn)	0.62		0.48

[†] Elemental composition of SRM 2975 particles has been reported by (Farahani et al., 2021).

Supplementary Table 2. Taq-Man probes for qRT-PCR.

Gene	Assay ID
Nlrp3	Mm00840904_m1 FAM MGB
Casp1	Mm00438023_m1 FAM MGB
Il1b	Mm00434228_m1 FAM MGB
Tnf	Mm00443258_m1 FAM MGB
Il6	Mm00446190_m1 FAM MGB
Ccl2	Mm00441242_m1 FAM MGB
Nos2	Mm00440502_m1 FAM MGB
Tgfb1	Mm01178820_m1 FAM MGB
Arg1	Mm00475988_m1 FAM MGB
Il10	Mm01288386_m1 FAM MGB

Supplementary Results

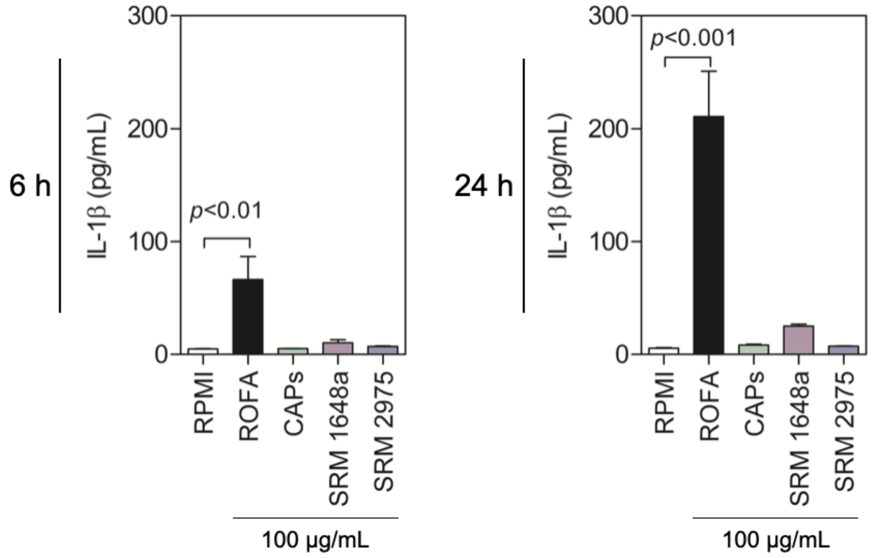

Supplementary Figure S1. IL-1β release in mice BMDMs incubated with $PM_{2.5}$. IL-1β levels were quantified by ELISA in cell culture supernatants from wild type BMDMs after incubation with $PM_{2.5}$ suspensions at 100 µg/mL for 6 or 24 hours.

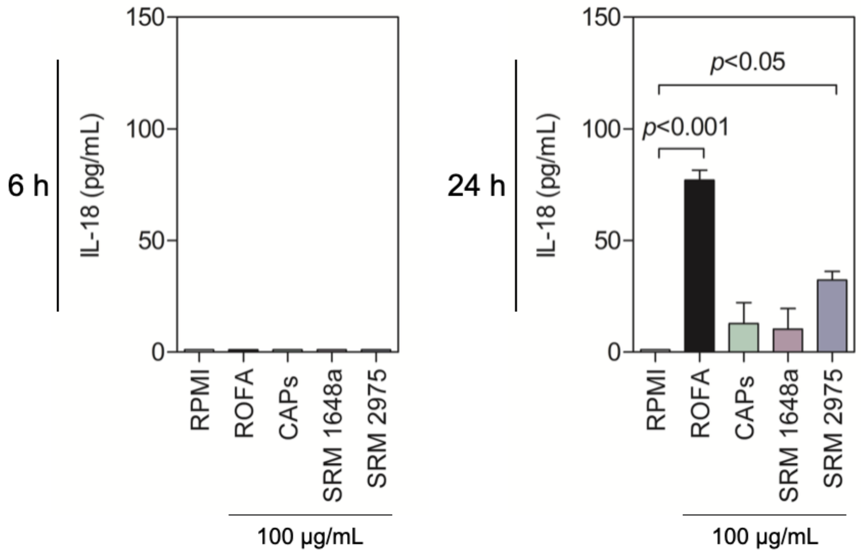

Supplementary Figure S2. IL-18 release in mice BMDMs incubated with $PM_{2.5}$. IL-18 levels were quantified by ELISA in cell culture supernatants from wild type BMDMs after incubation with $PM_{2.5}$ suspensions at 100 µg/mL for 6 or 24 hours.

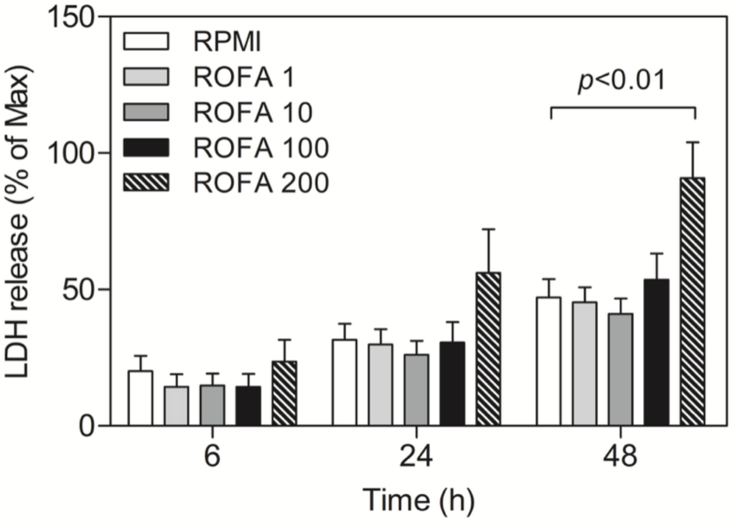

Supplementary Figure S3. Cell viability of mice BMDMs incubated with ROFA. LDH release was quantified by the CytoTox 96 assay in cell culture supernatants from wild type BMDMs after ROFA incubation at 1, 10, 100, or 200 µg/mL for 6, 24, and 48 hours.

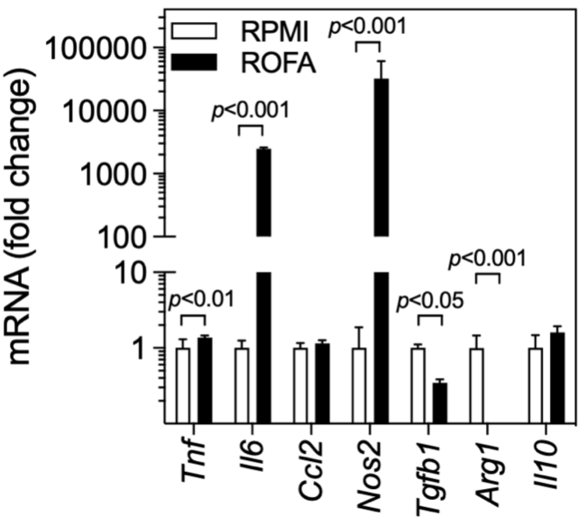

Supplementary Figure S4. Macrophage phenotype in mice BMDMs incubated with ROFA. Gene expression was assessed by qRT-PCR in wild type BMDMs incubated with ROFA at 100 µg/mL for 6 hours.

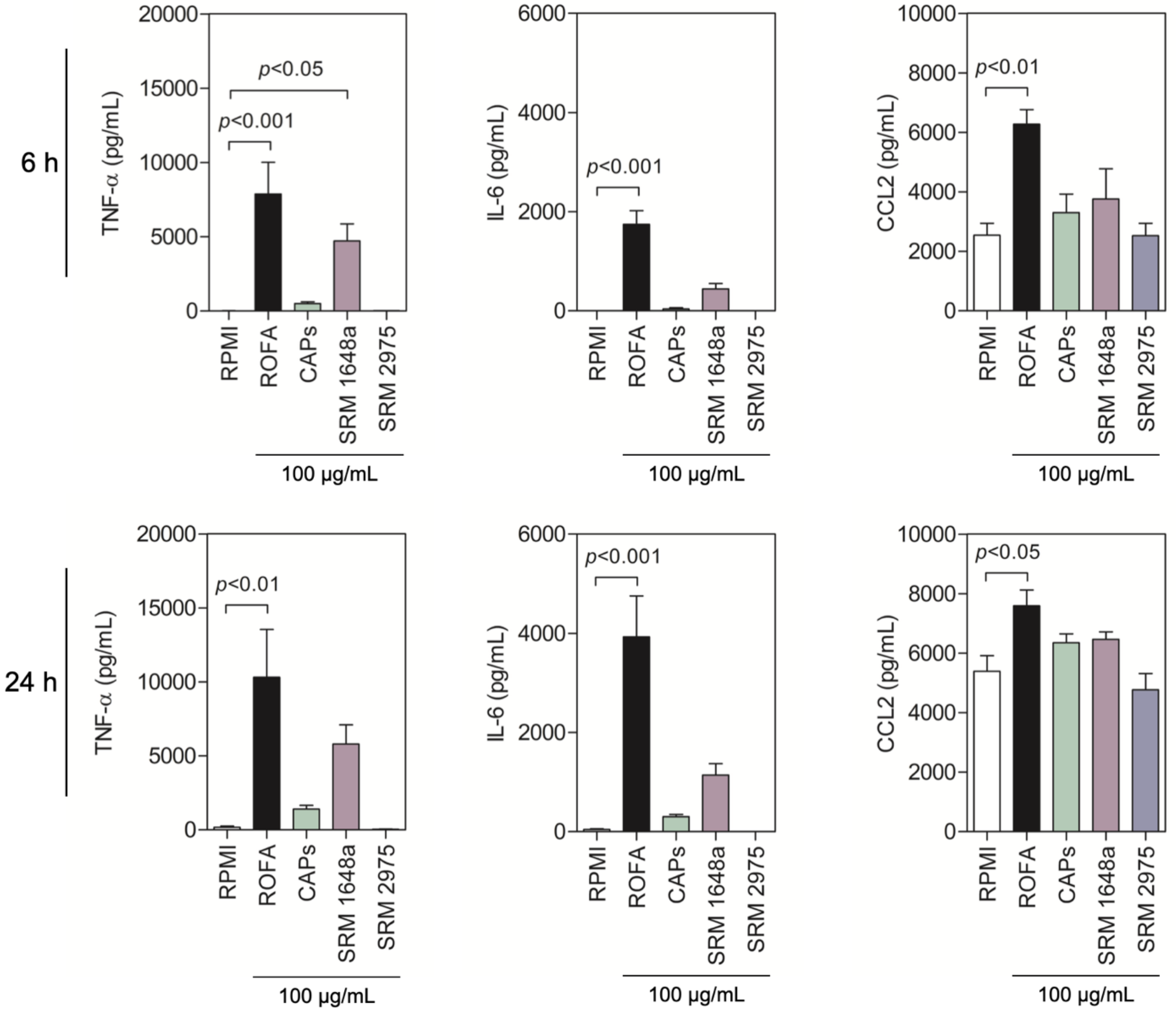

Supplementary Figure S5. Cytokine production in mice BMDMs incubated with $PM_{2.5}$. Cytokine levels were quantified by the CBA Mouse Inflammation Kit in cell culture supernatants from wild type BMDMs after incubation with $PM_{2.5}$ suspensions at 100 µg/mL for 6 or 24 hours.

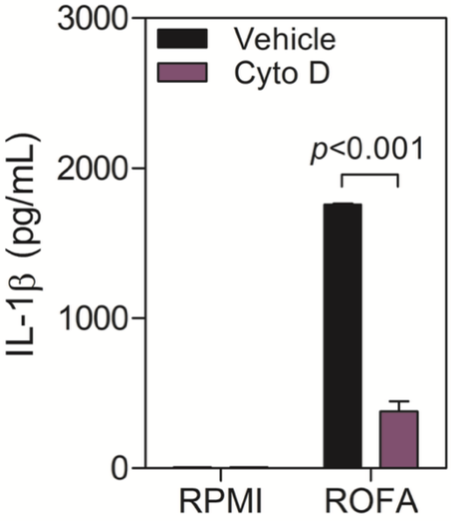

Supplementary Figure S6. Phagocytosis inhibition in mice BMDMs incubated with ROFA. IL-1β levels were quantified by ELISA in cell culture supernatants from wild type BMDMs preincubated with the phagocytosis inhibitor Cytochalasin D (CytoD) followed by incubation with ROFA at 100 μg/mL for 24 hours.

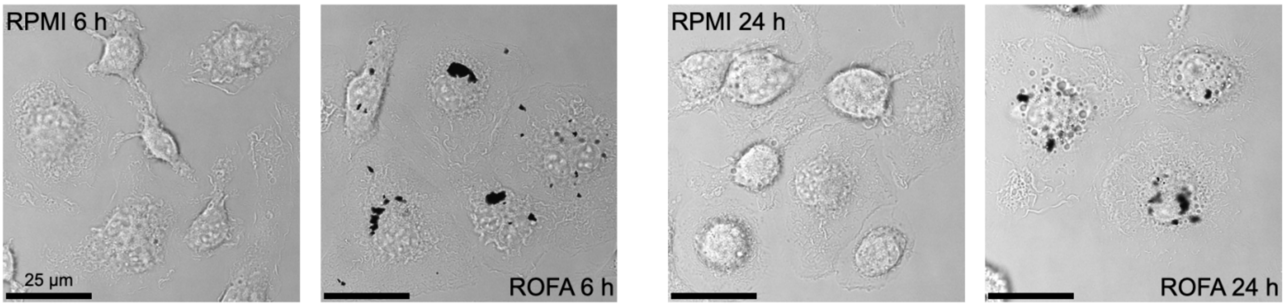

Supplementary Figure S7. $PM_{2.5}$ uptake by mice BMDMs. Wild type BMDMs were incubated with ROFA at 100 µg/mL or RPMI for 6 or 24 hours. Bright field pictures were acquired with a Leica SP8 confocal microscope equipped with a 63×/1.40 oil objective. Engulfed $PM_{2.5}$ can be visualized as black particulate material inside the cell cytoplasm in ROFA-exposed BMDMs.

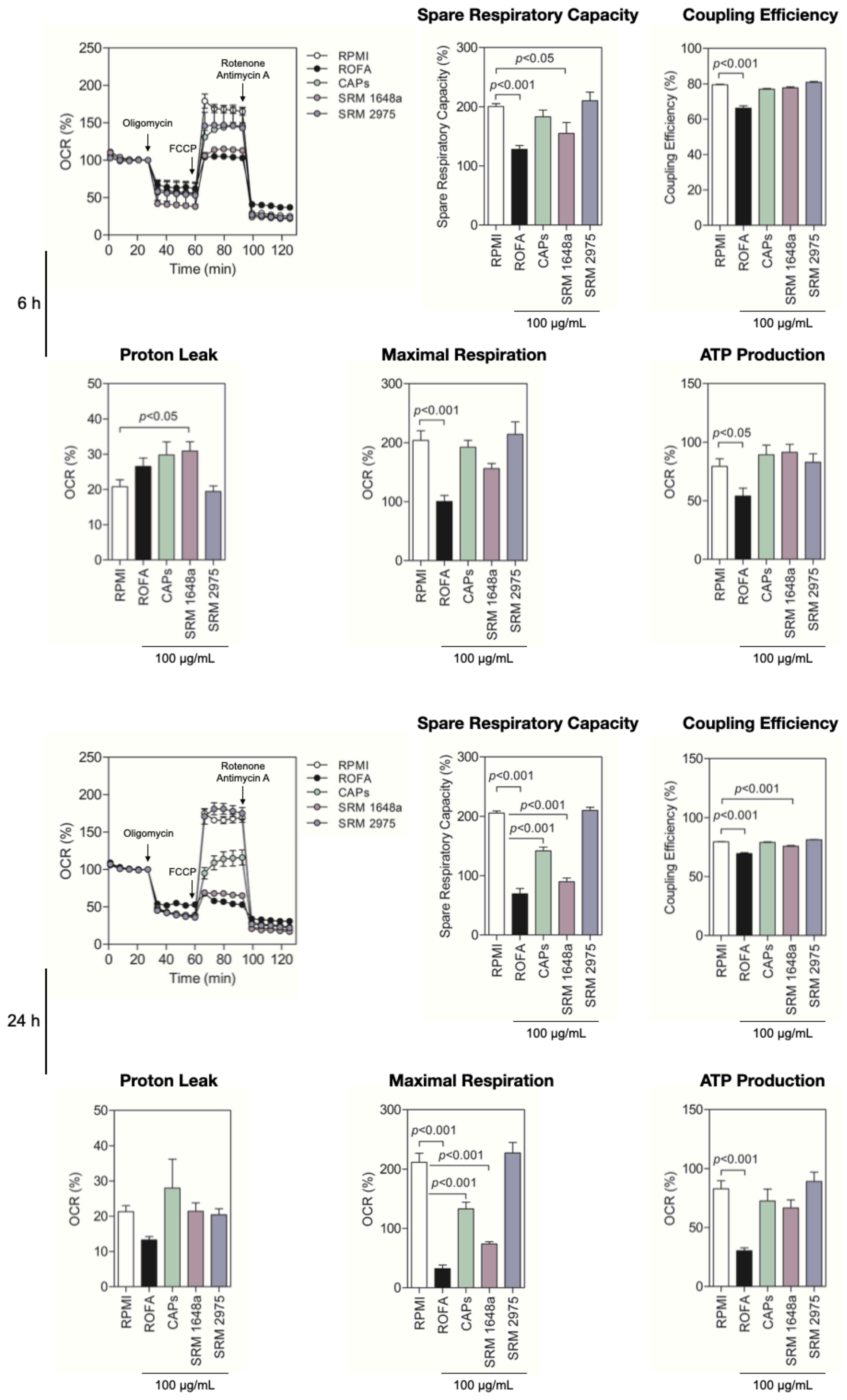

Supplementary Figure S8. Mitochondrial function in mice BMDMs incubated with $PM_{2.5}$. Mitochondrial oxygen consumption rate (OCR) was assessed by the Seahorse MitoStress Test in wild type BMDMs after incubation with $PM_{2.5}$ suspensions at 100 µg/mL for 6 or 24 hours. Indicators of mitochondrial bioenergetics were calculated from the OCR traces by the Seahorse XF Cell Mito Stress Test Report Generator Software.

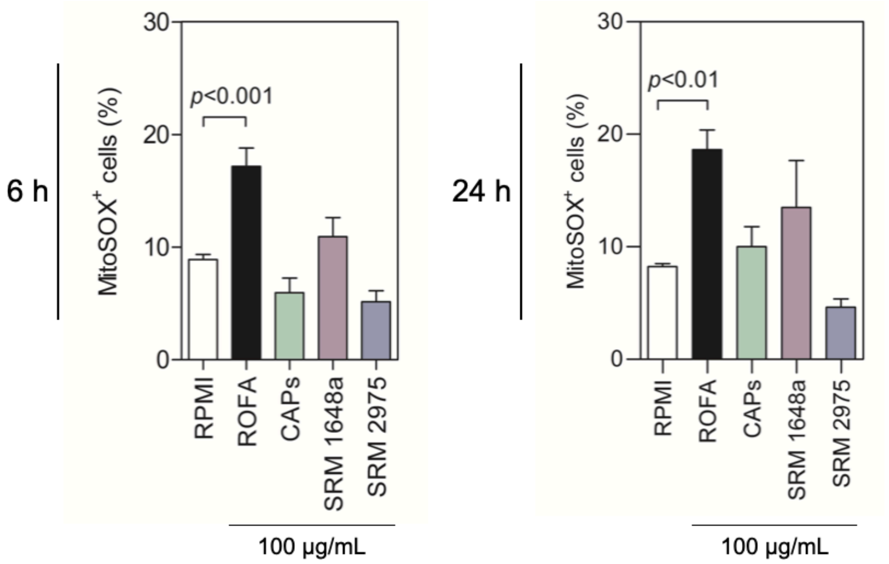

Supplementary Figure S9. Mitochondrial $O_2^{\bullet -}$ production in mice BMDMs incubated with $PM_{2.5}$. Quantification of $MitoSOX^+$ cells are indicative of mitochondrial $O_2^{\bullet -}$ production in wild type BMDMs after incubation with $PM_{2.5}$ suspensions at 100 µg/mL for 6 or 24 hours.

Supplementary References

Antunes, F., Cadenas, E., Brunk, U.T., 2001. Apoptosis induced by exposure to a low steady-state concentration of H2O2 is a consequence of lysosomal rupture. Biochem J 356, 549-555.

Brand, M.D., Nicholls, D.G., 2011. Assessing mitochondrial dysfunction in cells. Biochem J 435, 297-312.

Caceres, L., Paz, M.L., Garces, M., Calabro, V., Magnani, N.D., Martinefski, M., Martino Adami, P.V., Caltana, L., Tasat, D., Morelli, L., Tripodi, V., Valacchi, G., Alvarez, S., Gonzalez Maglio, D., Marchini, T., Evelson, P., 2020. NADPH oxidase and mitochondria are relevant sources of superoxide anion in the oxinflammatory response of macrophages exposed to airborne particulate matter. Ecotoxicol Environ Saf 205, 111186.

Farahani, V.J., Pirhadi, M., Sioutas, C., 2021. Are standardized diesel exhaust particles (DEP) representative of ambient particles in air pollution toxicological studies? Sci Total Environ 788, 147854.

Gross, C.J., Mishra, R., Schneider, K.S., Medard, G., Wettmarshausen, J., Dittlein, D.C., Shi, H., Gorka, O., Koenig, P.A., Fromm, S., Magnani, G., Cikovic, T., Hartjes, L., Smollich, J., Robertson, A.A.B., Cooper, M.A., Schmidt-Supprian, M., Schuster, M., Schroder, K., Broz, P., Traidl-Hoffmann, C., Beutler, B., Kuster, B., Ruland, J., Schneider, S., Perocchi, F., Gross, O., 2016. K(+) Efflux-Independent NLRP3 Inflammasome Activation by Small Molecules Targeting Mitochondria. Immunity 45, 761-773.

Hoss, F., Rolfes, V., Davanso, M.R., Braga, T.T., Franklin, B.S., 2018. Detection of ASC Speck Formation by Flow Cytometry and Chemical Cross-linking. Methods Mol Biol 1714, 149-165.

Magnani, N.D., Muresan, X.M., Belmonte, G., Cervellati, F., Sticozzi, C., Pecorelli, A., Miracco, C., Marchini, T., Evelson, P., Valacchi, G., 2016. Skin Damage Mechanisms Related to Airborne Particulate Matter Exposure. Toxicol Sci 149, 227-236.

Rog-Zielinska, E.A., Johnston, C.M., O'Toole, E.T., Morphew, M., Hoenger, A., Kohl, P., 2016. Electron tomography of rabbit cardiomyocyte three-dimensional ultrastructure. Prog Biophys Mol Biol 121, 77-84.

Rog-Zielinska, E.A., Scardigli, M., Peyronnet, R., Zgierski-Johnston, C.M., Greiner, J., Madl, J., O'Toole, E.T., Morphew, M., Hoenger, A., Sacconi, L., Kohl, P., 2021. Beat-by-Beat Cardiomyocyte T-Tubule Deformation Drives Tubular Content Exchange. Circ Res 128, 203-215.

Schneider, K.S., Thomas, C.J., Gross, O., 2013. Inflammasome activation and inhibition in primary murine bone marrow-derived cells, and assays for IL-1alpha, IL-1beta, and caspase-1. Methods Mol Biol 1040, 117-135.

SRM 1648a Certificate of Analysis https://tsapps.nist.gov/srmext/certificates/1648a.pdf

SRM 2975 Certificate of Analysis https://tsapps.nist.gov/srmext/certificates/2975.pdf
